# Breaking the vicarious trap: two buffers against close others’ environmental influence

**DOI:** 10.1186/s40359-026-05363-y

**Published:** 2026-10-02

**Authors:** Xiao Xiao, Youlong Zhan, Yiping Zhong

**Affiliations:** 1 Department of Psychology, Hunan First Normal University, Changsha, 410205 China; 2 Department of Psychology, Hunan University of Science and Technology, Xiangtan, 411201 China; 3 Department of Psychology, School of Education Science, Hunan Normal University, Changsha, 410081 China

**Keywords:** Pro-environmental behavior, Vicarious licensing and identification, Situational openness, Environmental self-identity, Moral regulation, Diary method

## Abstract

**Background:**

Close others' pro-environmental or anti-environmental behaviors can create a vicarious trap: good deeds trigger licensing (reducing one's own effort), and bad deeds induce identification (justifying inaction). However, how this trap can be mitigated remains unclear.

**Methods:**

Across four studies (total *N* = 523), we found evidence for the vicarious trap in pro‑environmental behavior (PEB) and tested two buffers: situational openness (Study 2) and environmental self‑identity (Studies 3–4). Study 1 compared relationship types; Study 4 combined a brief identity‑enhancement intervention with a 30‑day diary.

**Results:**

Study 1 (*N* = 97) showed that recalling close friends' (but not family members') environmental actions reduced subsequent PEB. Study 2 (*N* = 180) demonstrated that situational openness—making the recalled behavior public—eliminated the trap. Study 3 (*N* = 180) showed that high environmental self-identity provided complete immunity. Study 4 (*N* = 66) provided preliminary, exploratory evidence that identity enhancement promotes an identity‑driven pathway: daily environmental self‑identity predicted next‑day PEB with autoregressive controls, and mediation analyses confirmed identity as the causal mechanism.

**Conclusions:**

These findings identify boundary conditions and provide initial evidence for an identity‑driven behavioral pathway, with practical implications for interventions leveraging social norms and self‑concept to promote sustained PEB.

**Supplementary Information:**

The online version contains supplementary material available at https://doi.org/10.1186/s40359-026-05363-y.

## Introduction

Daily environmental decisions reveal a thought-provoking phenomenon. Imagine learning that your romantic partner maintains a vegetarian diet to reduce their carbon footprint. Do you unconsciously think, "Since they are being so environmentally responsible, I can occasionally choose high-carbon transportation without guilt?" Conversely, upon discovering that family members frequently use disposable plastics, do you rationalize, "If my family behaves this way, my waste-sorting efforts seem futile?" These subtle processes illustrate a *vicarious trap* created by close relationships in the environmental domain. Close others' pro-environmental behaviors (PEB) may trigger v*icarious licensing* (i.e., "because they did it, I do not have to"), reducing one's own efforts [25],their anti-environmental behaviors may induce *vicarious identification* (i.e., "because they did it, I can too"), justifying inaction [20]. Together, these mechanisms form a self-indulgent cycle that threatens sustained PEB. Understanding how this *vicarious trap* can be mitigated is therefore of both theoretical and practical importance.

### Theoretical foundation: vicarious moral self-regulation

The *vicarious trap* is grounded in vicarious moral self-regulation theory—a theoretical framework that extends traditional moral self-regulation theory [32, 52] from the intrapersonal to the interpersonal domain [10]. Traditional moral self-regulation posits that individuals maintain a stable moral self-image through internal balancing: past moral behavior licenses subsequent indulgence (moral licensing), while past immoral behavior motivates compensatory good deeds (moral cleansing). Vicarious moral self-regulation theory argues that this balancing mechanism can be socially outsourced: individuals can regulate their moral self-image not only through their own actions but also through the actions of close others [29, 48]. Observing a close other ‘s PEB reduces one’s own subsequent PEB (vicarious licensing, [20, 25],observing a close other ‘s anti-environmental behavior makes inaction seem justifiable (vicarious identification (also termed vicarious moral disengagement or moral contagion, [18, 19, 21, 40],and compensatory responses (vicarious cleansing) are often absent, particularly in the environmental domain [16].

Two complementary theoretical frameworks—vicarious self-perception theory and self-other overlap theory—jointly explain the underlying mechanisms of vicarious moral self-regulation. Vicarious self-perception theory [20] extends traditional self-perception theory [3] by proposing that individuals can infer their own attributes not only from observing their own freely chosen actions, but also from observing the freely chosen actions of others—almost as if they had observed themselves performing the acts. When a person observes a close other's freely chosen behavior, they incorporate attributes relevant to that behavior into their own self-concept, and these changes in self-perception subsequently guide their own behavior. However, this vicarious self-perception process does not occur for all others,it requires psychological closeness. Self-other overlap theory [1] specifies this condition: close relationships blur the boundaries between self and other, such that the traits, behaviors, and characteristics of intimate others are perceived, in part, as one's own. Neurophysiological evidence confirms that the degree of such overlap modulates how deeply others' actions are processed as self-relevant [14, 50].

Integrating these two theoretical foundations, vicarious moral self-regulation operates as follows: vicarious self-perception explains why others' actions can influence one's own moral calculus—because their freely chosen behaviors are incorporated into one's self-concept; self-other overlap explains who exerts this influence—those with whom one shares the greatest psychological overlap (e.g., loved ones and close friends). A critical distinction is necessary. Although both vicarious licensing and vicarious identification share the common precondition of self-other overlap, they operate through divergent psychological routes. Vicarious licensing occurs because a close other's pro-environmental act creates shared moral credit, allowing the individual to conclude that sufficient environmental effort has been made on their behalf, thereby licensing personal inaction. Vicarious identification occurs because a close other's anti-environmental act normalizes the behavior, leading the individual to infer that such conduct is acceptable and to engage in similar actions. Both processes converge on the same behavioral outcome—reduced PEB—through distinct mechanisms: credit-sharing versus norm-inference.

Importantly, the strength of these vicarious effects may critically depend on the developmental stage of the individual. While family exerts a powerful influence during childhood and adolescence [13], emerging adulthood (ages 18–25) is characterized by a developmental shift toward peer and romantic relationships [12]. During this period, peers become the primary reference group for norm formation and identity exploration [7]. Therefore, the vicarious trap should be more strongly activated by psychologically close peers (i.e., loved ones) than by family members, as the former represent the most salient source of social influence in this specific developmental context. Thus, Hypothesis 1: Recalling a close friend's or loved one's (but not a family member's) PEB or anti-environmental behavior will reduce subsequent PEB compared to a neutral control. For family members, these effects will be weaker due to lower self-other overlap in emerging adulthood. Having established the existence and mechanisms of the vicarious trap, a critical question arises: Can this trap be broken? The following sections identify two potential buffers—one external (situational openness) and one internal (environmental self-identity)—that may attenuate or eliminate vicarious licensing and identification effects.

### Situational openness as an external buffer

Situational openness—the degree to which behavior is observable by others—can attenuate vicarious licensing and identification. This buffering effect operates primarily through a shift in normative inference rather than through impression management per se. When a friend's (anti-)environmental behavior is made public, it is transformed from a private anecdote into a social fact, thereby making descriptive and injunctive social norms [9, 36] more salient. This powerful normative information overrides the private moral rationalizations (licensing and identification), guiding behavior even in a subsequently private task. Conversely, anonymity facilitates selfish or immoral behavior [4, 5, 51]. Critically, even when only the recalled behavior of a close other is made public (rather than the participant's own behavior), this situational openness activates salient social norms and self-presentation concerns, thereby influencing subsequent private choices [2]. The term 'situational openness' is used consistently throughout this manuscript to refer to this construct, operationalized as whether the recalled behavior of a close friend is disclosed to others or kept anonymous. Thus, Hypothesis 2 was proposed that the vicarious trap would be attenuated or eliminated under conditions of high situational openness (situational openness) compared to low situational openness (anonymous context), such that recalling a close friend's environmental behavior would not significantly reduce PEB when that behavior was made public.

### Environmental self-identity as an internal buffer

Environmental self-identity refers to the extent to which individuals see themselves as environmentally friendly persons [44]. According to self-concept theory, when pro-environmental values become central to self-identity, behavioral decisions are guided by stable internal standards rather than by social comparisons or situational cues [47]. For high-identity individuals, the question "Should I act pro-environmentally?" is already answered by the self-definition "I am an environmentally friendly person." This internal anchor should render external moral information—whether a friend's virtue or vice—irrelevant to behavioral decisions [17]. Thus, Hypothesis 3 was proposed that: High environmental self-identity will buffer against the vicarious trap, such that recalling a close friend's environmental behavior will not affect PEB among high-identity individuals.

Critically, environmental self-identity may not be merely a static buffer but a dynamic force capable of initiating a positive feedback loop. Drawing on self-perception theory [3], engaging in PEB can reinforce one's environmental self-identity, which in turn motivates future PEB. This hypothesized bidirectional cycle—where identity and behavior reciprocally enhance each other—could form the foundation for a sustainable escape from the vicarious trap. Study 4 is designed to provide an initial, ecologically valid test of this dynamic spiral in daily life. For those who deeply identify as an “environmentally friendly person”, their behavior is guided by an internal compass [47]. A friend's behavior, whether good or bad, simply does not provide relevant moral information,it is like water off a duck's back. The question of “what should I do?” is already answered by their core self-definition, making them naturally immune to the vicarious influence of others. Hence, Hypothesis 4 was proposed that: Compared with individuals low in environmental self-identity, those high in environmental self-identity will better resist the *vicarious trap* induced by close others and will consistently engage in more PEBs.

### The current study

Although prior work has demonstrated vicarious regulation in moral domains, gaps remain regarding PEB. First, studies have not systematically compared effects across different types of close relationships. Second, effective pathways to mitigate vicarious traps are underexplored. Third, while environmental self-identity has been shown to buffer the trap in cross-sectional designs, it remains unknown whether identity can be cultivated to produce lasting protection and whether identity and behavior dynamically influence each other in daily life. To address these gaps, four studies were conducted. Study 1 examined whether the vicarious trap varies by relationship type (family vs. loved ones). To provide a comparative benchmark, a self-recall condition was included, which allowed comparison of the magnitude and direction of vicarious effects (triggered by recalling close others' behavior) against those of traditional moral self-regulation effects (triggered by recalling one's own behavior). Study 2 tested situational openness as an external buffer. Study 3 tested environmental self-identity as an internal buffer. Study 4 extended these experimental findings to naturalistic settings by combining a brief identity-enhancement intervention with a 30-day daily diary. This design allowed examination of whether environmental self-identity is malleable and causally protective, and whether daily identity and PEB frequency show bidirectional associations over time using mixed-effects models with autoregressive controls. By integrating controlled experiments with longitudinal tracking, the present research aims to provide both internal and ecological validity for understanding how the trap can be broken.

## Study 1

### Methods

#### Participants

 G*Power 3.1 indicated that a minimum of 84 participants were needed to detect a medium effect (*f* = 0.30) with power *β* = 0.85. A total of 97 Chinese college students were recruited (46 male, 51 female; age 18–22 years, *M* = 20.18, *SD* = 1.45). All lived in school dormitories, were right‑handed, had no history of psychiatric illness, and had normal or corrected‑to‑normal vision. Participants were randomly assigned to one of three recall‑behavior groups: environmentally friendly (*n* = 30), unfriendly (*n* = 34), or neutral (*n* = 33). All provided informed consent and received compensation based on their performance. The study was approved by the Academic Ethics Committee of Hunan Normal University.

#### Design

A 3 (recall behavior: environmentally friendly, unfriendly, neutral) × 3 (recall object: family member, loved one, self) mixed design was used, with recall behavior as a between-subjects factor and recall object as a within-subjects factor. A self-recall condition was included to provide a comparative benchmark for the magnitude of vicarious effects relative to traditional moral self-regulation effects—that is, to assess whether observing a close other's environmental behavior produces effects comparable in magnitude to recalling one's own behavior. Dependent variables were intentions to engage in PEB and proportion of carbon–neutral choices.

#### Materials and tasks

##### Inclusion of Others in the Self (IOS) Scale

The IOS [1] consists of seven pairs of overlapping circles representing degrees of closeness. Before the experiment, participants nominated a same‑sex close friend and a family member, and rated closeness on the IOS. A score ≥ 5 (for friend) or ≥ 4.5 (for family) was required to meet the operational definition of intimate other,if not, participants nominated another person until criteria were met.

##### Event recall and description task

Adapted from Sachdeva et al. [38], participants were instructed to recall and describe in at least 100 words a specific behavior by the target person within the past six months. The friendly group recalled a pro‑environmental action, the unfriendly group recalled an anti‑environmental action, and the neutral group recalled an action unrelated to the environment (e.g., daily routine). After writing, participants rated the environmental friendliness of the recalled behavior (1 = very unfriendly, 7 = very friendly) as a manipulation check.

##### Intentions to engage in PEB

This six‑item scale [31],α = 0.91) measured intentions to engage in various PEBs (e.g., signing a petition) on a 7‑point Likert scale (1 = completely disagree, 7 = completely agree).

##### Carbon emission task

**Fig. 1:**
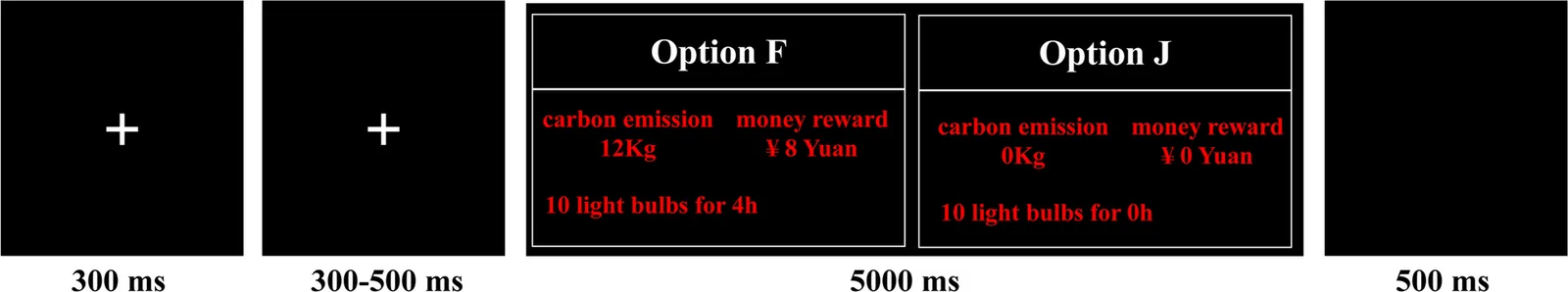
The sequence of events in the carbon emissions for monetary rewards task

Following Berger and Wyss [6] and Xiao et al. [48], participants completed 10 trials pitting monetary gain against carbon emissions. In each trial, they chose between option F (financial reward, generates CO₂) and option J (no reward, no emissions). Monetary amounts (¥2, ¥4, ¥6, ¥8, ¥10) and corresponding emissions (3, 6, 9, 12, 15 kg) were paired to create four conditions: low-emissions-low-benefit, high-emissions-low-benefit, low-emissions-high-benefit, and high-emissions-high-benefit, with ¥6/9 kg as an intermediate reference. The dependent variable was the proportion of trials in which participants chose the carbon–neutral option (Option J) out of 10 trials. One trial was randomly selected for payment,the money was deducted from the base ¥30 compensation, and the corresponding carbon emissions were ostensibly realized by lighting bulbs for different durations (Fig. 1).

#### Procedure

First, participants nominated a family member and a loved one (romantic partner or closest friend) and completed the IOS for each. To control for pre‑existing environmental attitudes, they completed the Environmental Self‑Identity Scale [44]. Then they performed the event recall task for each target in pseudo‑random order, followed by the PEB intentions scale and the carbon emission task. Finally, one trial was randomly selected for payment. The experiment lasted about 30 min. All study materials, anonymized data, analysis code, and codebook can be accessed via the following anonymized OSF link (https://osf.io/dtqyj/overview?view_only=915fe15173cd4ce49d3ecc82356d3f96).

## Results

### Manipulation check

IOS scores were higher for loved ones (*M* = 5.68, *SD* = 0.79) than for family members (*M* = 4.86, *SD* = 0.83), *t*_(96)_ = 7.10, *p* < 0.001, indicating that the manipulation of recall object was effective. Regarding environmental behavior attributes for the act of reminiscing, in the environmentally friendly, unfriendly, and neutral behavior groups, all participants recalled behaviors as environmentally friendly (4.23 ± 0.93), environmentally unfriendly (1.63 ± 0.85), and unrelated to environmental behavior in their daily lives, respectively. These results confirmed the effectiveness of the free-recall task. Additionally, no significant differences were observed in environmental self-identity among the three groups (environmentally friendly: 4.95 ± 1.22; unfriendly: 5.02 ± 1.12; neutral: 4.86 ± 1.56), *F*_(2, 94)_ = 1.03, *p* = 0.31 (see Table 1).

**Table 1:** Results of IOS scores, environmental ratings, pro-environmental intentions, and green purchasing behaviors in Study 1 (M ± SD)

Variables	Recalling objects	Environmentally friendly group (*n* = 30)	Environmentally unfriendly group (*n* = 34)	Environmentally neutral group (*n* = 33)
Scores of IOS	family members	4.63 ± 0.81	5.03 ± 0.80	4.91 ± 0.84
	loved ones	5.50 ± 0.78	5.71 ± 0.87	5.82 ± 0.88
Environmental ratings	family members	4.11 ± 0.52	1.46 ± 0.45	/
	loved ones	4.36 ± 0.46	1.83 ± 0.42	/
	oneself	4.22 ± 0.41	1.61 ± 0.46	/
Pro-environmental intentions	family members	5.16 ± 0.86	4.79 ± 1.18	4.67 ± 1.35
	loved ones	5.13 ± 0.89	4.77 ± 1.15	4.63 ± 1.46
	oneself	5.07 ± 0.88	4.94 ± 1.03	4.63 ± 1.28
Carbon neutral behavior	family members	0.65 ± 0.19	0.61 ± 0.20	0.66 ± 0.17
	loved ones	0.56 ± 0.14	0.57 ± 0.19	0.73 ± 0.16
	oneself	0.57 ± 0.15	0.58 ± 0.17	0.72 ± 0.15

### PEB intentions and carbon neutral behavior

Regarding PEB intentions, the main effects of group (environmentally friendly: 5.12 ± 0.85, unfriendly: 4.83 ± 1.10, neutral: 4.64 ± 1.34), *F*_(2, 94)_ = 1.47, *p* = 0.24, *η*_*p*_^*2*^ = 0.03 and recall object were not significant (family member: 4.86 ± 1.16, loved one: 4.84 ± 1.20, self: 4.88 ± 1.09), *F*_(2, 188)_ = 0.41, *p* = 0.66, *η*_*p*_^*2*^ = 0.01. Furthermore, the interaction between group and recall object was not significant, *F*_(4, 188)_ = 2.04, *p* = 0.09, *η*_*p*_^*2*^ = 0.04 (see Table 1).

**Fig. 2:**
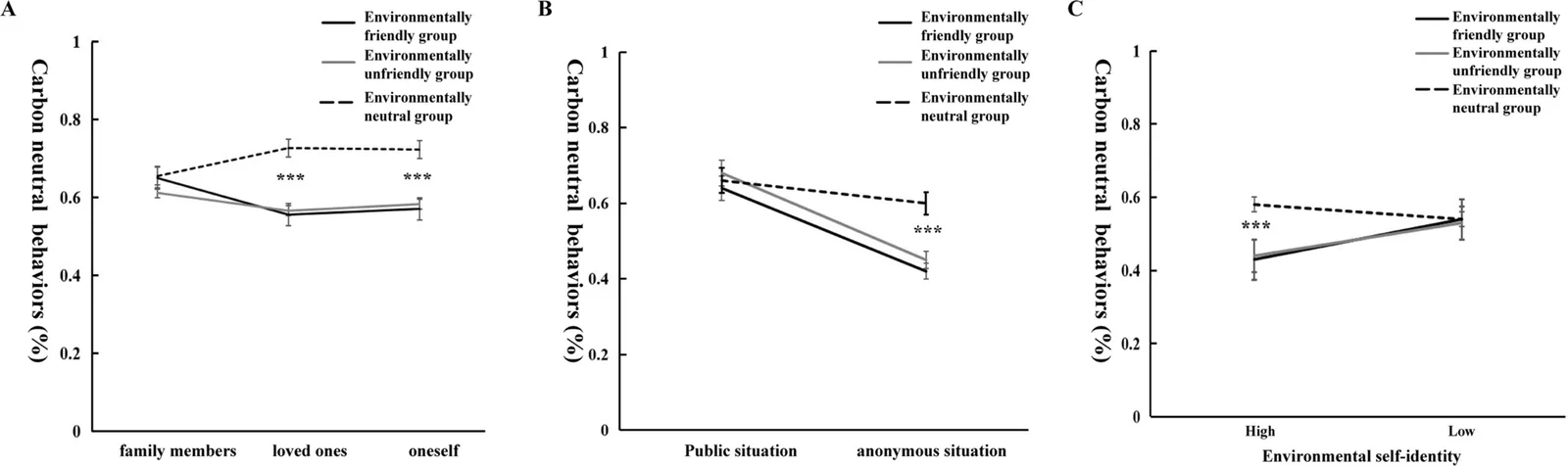
Proportions of carbon neutral behaviors under different conditions in Study 1 (Panel **A**, 2 (Panel **B**), and 3 (Panel **C**). Notes: ^∗^*P* < 0.05, ^∗∗^*P* < 0.01, ^∗∗∗^*P* < 0.001

Based on carbon neutral behavior, the main effect of group was significant, *F*_(2, 94)_ = 7.68,* p* = 0.001, *η*_*p*_^*2*^ = 0.14; multiple comparisons revealed that both the environmentally friendly (0.59 ± 0.12, *t*_(61)_ = −3.74, *p* < 0.001) and unfriendly groups (0.59 ± 0.16, *t*_(65)_ = −3.41, *p* = 0.001) exhibited significantly lower levels of carbon neutral behavior compared with the environmentally neutral group (0.70 ± 0.11), demonstrating pronounced *vicarious licensing* and *identity* effect; however, no significant difference was observed between the environmentally friendly (0.59 ± 0.12) and unfriendly group (0.59 ± 0.16, *t*_(62)_ = 0.15, *p* = 0.88). Moreover, the main effect of recall object was not significant, *F*_(2, 188)_ = 0.78, *p* = 0.46, *η*_*p*_^*2*^ = 0.01. However, the interaction between group and recall object was significant, *F*_(4, 188)_ = 4.22, *p* = 0.003, *η*_*p*_^*2*^ = 0.08. Simple effect analyses revealed that when recalling the behaviors of either the self (0.72 ± 0.15) or loved ones (0.73 ± 0.16), both the environmentally friendly (the self: 0.57 ± 0.15, *t*_(61)_ = −4.08, *p* < 0.001; loved ones: 0.56 ± 0.14, *t*_(61)_ = −4.51, *p* < 0.001) and unfriendly groups (the self: 0.58 ± 0.17, *t*_(65)_ = −3.58, *p* = 0.001; loved ones: 0.57 ± 0.19, *t*_(65)_ = −3.72, *p* < 0.001) exhibited significantly lower levels of carbon neutral behavior (the self: *F*_(2, 94)_ = 9.51, *p* < 0.001, *η*_*p*_^*2*^ = 0.17; loved ones: *F*_(2, 94)_ = 10.79, *p* < 0.001, *η*_*p*_^*2*^ = 0.19) compared with the environmentally neutral group. However, when participants recalled the behaviors of family members, no significant differences were observed among the three groups (environmentally friendly: 0.65 ± 0.19, unfriendly: 0.61 ± 0.20, neutral: 0.66 ± 0.17), *F*_(2, 94)_ = 0.54, *p* = 0.59, *η*_*p*_^*2*^ = 0.01 (see Table 1 and Fig. 2A).

### Discussion of Study 1

Study 1 demonstrated that recalling a loved one's pro-environmental or anti-environmental behavior reduced participants' own PEB, providing initial evidence for a *vicarious trap* characterized by both *licensin*g and i*dentification* effects. The absence of *vicarious cleansing* confirms an asymmetry in environmental moral regulation [29]. Notably, these effects were absent when recalling family members' behaviors, suggesting that psychological closeness, rather than kinship, determines susceptibility to vicarious influence during emerging adulthood. These findings establish the phenomenon but leave open the question of how the trap can be disrupted. An interesting discrepancy emerged in Study 1: the *vicarious trap* was clearly observed in the carbon emission task but not in the self-reported PEB intentions scale. This discrepancy may reflect the differential sensitivity of the two measures. The PEB intentions scale, being explicit and self-reported, is more susceptible to social desirability bias [35], potentially leading participants to consciously 'correct' their responses to appear more environmentally responsible after recalling a close other's behavior. In contrast, the carbon emission task is a behavioral proxy with real monetary and environmental consequences, making it less prone to such explicit self-presentation strategies. It is therefore a more sensitive and valid measure of the subtle, often automatic, process of vicarious moral regulation [6], [48]. This finding underscores the importance of using behavioral measures in future research on this topic.

## Study 2

### Methods

#### Participants

G*Power indicated a minimum of 125 participants for medium effect (*f* = 0.30) with power 0.85. A total of 180 Chinese college students were recruited (73 male, 107 female; *M *_*age*_ = 19.64, *SD* = 1.24) living in dormitories, with average close‑friendship duration 17.68 months. They were randomly assigned to a 3 (recall behavior: friendly, unfriendly, neutral) × 2 (situation: anonymous, public) between‑subjects design.

#### Materials and procedure

The procedure was similar to Study 1 except that participants recalled only a close same‑sex friend (IOS ≥ 5). After the recall task, the situational openness manipulation was introduced [33]. In the public condition, participants were told: “The environmental behaviors you recalled are very typical and representative. These examples illustrate for future participants who will perform the recall task.” They then provided personal information (name, phone, gender) ostensibly for this purpose. In the anonymous condition, they were told that no personal information would be collected during the experiment (data were linked via anonymous codes later). After the manipulation, participants completed the pro‑environmental attitude scale [27],4 items, e.g., “The Green Spring Festival is a good way to reduce resource consumption”,α = 0.85) and the carbon emission task. Order was counterbalanced. In addition, all study materials and anonymized data, can be accessed via the following anonymized OSF link (https://osf.io/dtqyj/overview?view_only=915fe15173cd4ce49d3ecc82356d3f96).

## Results

### Manipulation check

For the IOS scores, students reported a high degree of overlap with close friends (5.36 ± 0.98). Regarding environmental attributes for the recalling, in the environmentally friendly behavior, unfriendly, and neutral group, all participants recalled behaviors as environmentally friendly (4.26 ± 1.21), unfriendly (1.69 ± 0.92), and unrelated to environmental behavior, respectively. These results confirmed the effectiveness of the free-recall task (see Table 2).

**Table 2:** Results of IOS scores, environmental ratings, pro-environmental attitude, and carbon neutral behaviors in Study 2 (M ± SD)

Variables	Situational Openness	Environmentally friendly group (*n* = 60)	Environmentally unfriendly group (*n* = 60)	Environmentally neutral group (*n* = 60)
Scores of IOS	Public situation (*n* = 30)	5.03 ± 1.10	5.63 ± 0.85	5.07 ± 0.87
	Anonymous situation (*n* = 30)	5.63 ± 1.07	5.40 ± 0.7	5.37 ± 0.93
Environmental ratings	Public situation (*n* = 30)	4.22 ± 1.31	1.56 ± 0.84	/
	Anonymous situation (*n* = 30)	4.31 ± 1.11	1.82 ± 1.04	/
Pro-environmental attitude	Public situation (*n* = 30)	5.86 ± 1.23	5.25 ± 1.23	5.76 ± 0.83
	Anonymous situation (*n* = 30)	5.36 ± 1.08	5.21 ± 1.29	5.03 ± 1.49
Carbon neutral behavior	Public situation (*n* = 30)	0.64 ± 0.17	0.68 ± 0.15	0.66 ± 0.16
	Anonymous situation (*n* = 30)	0.42 ± 0.16	0.45 ± 0.13	0.60 ± 0.21

### Pro-environmental attitude and carbon neutral behavior

Regarding pro-environmental attitude, the main effect of group was not significant (environmentally friendly: 5.23 ± 1.45; unfriendly: 5.61 ± 1.18; neutral: 5.39 ± 1.25), *F*_(2,174)_ = 1.32, *p* = 0.27, *η*_*p*_^*2*^ = 0.02. However, the main effect of situation was significant, *F*_(1, 174)_ = 4.93, *p* = 0.03, *η*_*p*_^*2*^ = 0.03, namely participants reported more positive pro-environmental attitudes in the public situation (5.62 ± 1.28) than in the anonymous situation (5.20 ± 1.29). The interaction between group and situation was not significant, *F*_(2, 174)_ = 1.13, *p* = 0.33, *η*_*p*_^*2*^ = 0.01 (see Table 2).

For carbon neutral behavior, the main effect of group was significant, *F*_(2, 174)_ = 5.85,* p* = 0.003, *η*_*p*_^*2*^ = 0.06; multiple comparisons revealed that compared with the neutral group (0.63 ± 0.19), the environmentally friendly (0.53 ± 0.20, *t*_(118)_ = −2.85, *p* = 0.005) and unfriendly (0.56 ± 0.18, *t*_(118)_ = −2.08, *p* = 0.04) behavior group exhibited significantly less carbon neutral behavior, indicating a clear *vicarious licensin*g and *identity* effect. However, no significant was observed difference between the environmentally friendly and unfriendly groups (*t*_(118)_ = −0.87, *p* = 0.39). The main effect of situation was significant, *F*_(1, 174)_ = 47.17,* p* = 0.001, *η*_*p*_^*2*^ = 0.21: participants reported more green purchasing behaviors in the public condition (0.66 ± 0.16) than in the anonymous condition (0.49 ± 0.19). Furthermore, their interaction was significant, *F*_(2, 174)_ = 5.12, *p* = 0.007, *η*_*p*_^*2*^ = 0.06. Simple effect analysis revealed that under the anonymous condition, carbon neutral behavior differed significantly among the three groups, *F*_(2, 175)_ = 8.37,* p* < 0.001; multiple comparisons revealed that compared with the neutral group (0.60 ± 0.21), both the environmentally friendly (0.42 ± 0.16, *t*_(58)_ = −3.74, *p* < 0.001) and unfriendly (0.45 ± 0.13, *t*_(58)_ = −3.46, *p* = 0.001) groups exhibited significantly less carbon neutral behavior, consistent with the *vicarious licensing* and *identity* effect; however, no significant differences was observed between the environmental friendly and unfriendly (*t*_(58)_ = −0.61, *p* = 0.55). Under the public condition, no significant differences were observed among the three groups (environmentally friendly: 0.64 ± 0.17, unfriendly: 0.68 ± 0.15, neutral: 0.66 ± 0.16), *F*_(2, 175)_ = 0.30,* p* = 0.74 (see Table 2 and Fig. 2B). In addiction, to rule out the alternative explanation that the buffering effect of the situational openness was merely due to a general elevation of baseline behavior, the 3 (Recall behavior) analysis was re-run × 2 (Situation) ANCOVA on carbon–neutral choices, with pro-environmental attitude as a covariate. The critical interaction remained significant, *F*_(2, 175)_ = 4.87, *p* = 0.009, *ηₚ*^*2*^ = 0.05, indicating that the buffering effect of the situational openness persists even after controlling for general shifts in environmental attitudes.

### Discussion of Study 2

Study 2 showed that making the recalled behavior public eliminated the *vicarious trap*, even though participants' own choices remained private. This demonstrates that situational openness serves as an external buffer. Consistent with the theoretical framing, this buffering effect likely operates through the activation of salient social norms rather than through impression management per se—when a friend's behavior is made public, it signals what is socially approved or disapproved within the reference group, establishing a normative standard that guides subsequent behavior even in private tasks [9]. The findings provide proof-of-concept that the social visibility of others' actions can override vicarious licensing and identification. Nevertheless, it is acknowledged that it is not possible to definitively rule out the ceiling effect explanation entirely,future research with within-subjects designs and repeated baseline measures would provide a more definitive test.

## Study 3

### Methods

#### Participants

A total of 485 college students were prescreened with the Environmental Self-Identity Scale [44]. Following the extreme-group method [24], the top and bottom 27% of scorers were selected as high- and low-identity groups. This approach increases statistical power for detecting interaction effects by maximizing between-group differences, which was necessary given the resource constraints of the laboratory-based carbon emission task. However, it may inflate effect size estimates relative to continuous-variable approaches and reduces generalizability to individuals with moderate identity levels. From each group, 90 participants were randomly invited, yielding a final sample of 180 (77 male,M age = 20.08, SD = 1.72,average friendship duratio*n* = 18.24 months).

#### Materials

**Environmental Self-Identity Scale.** The Environmental Self-Identity Scale developed by Van der Werff et al. [44] was adopted. It comprised 3 items (such as "Taking environmental issues into account when doing things is an important characteristic of my personal identity") rated on a 7-point scale (1 = complete disagreement, 7 = complete agreement). It has been widely used among Chinese participants, and has an internal consistency coefficient of 0.90.

#### Procedure

First, the participants nominated one same-sex close friend and completed the IOS scale, similar to in Experiment 1. Subsequently, they completed the operational check for the recalling task. Then they completed the pro-environmental attitude scale and carbon neutral task. The entire experiment lasted approximately 30 min, and a certain amount of compensation was provided. In addition, all study materials and anonymized data, can be accessed via the following anonymized OSF link (https://osf.io/dtqyj/overview?view_only=915fe15173cd4ce49d3ecc82356d3f96).

## Results

### Manipulation check

Regarding environmental self-identity, compared with those with low environmental self-identity (3.42 ± 0.81), college students with high environmental self-identity reported a higher level of environmental self-identity (5.93 ± 0.51), *t* = 25.05, *p* < 0.001. No significant main effects of group were observed (*F*_(2, 174)_ = 1.06,* p* = 0.35, *η*_*p*_^*2*^ = 0.01). Furthermore, results indicated that grouping based on environmental self-identity was effective. For IOS scores, college students reported a high degree of overlap with close friends (5.22 ± 0.86), indicating that the close friends nominated by participants met the experimental criteria. Regarding environmental ratings for the recalling, in the environmentally friendly, unfriendly, and neutral behavior group, all participants recalled behaviors as environmentally friendly (4.67 ± 1.35), unfriendly (1.63 ± 0.87), and unrelated to environmental behavior, respectively. These results confirmed the effectiveness of the free-recall task (see Table 3).

**Table 3:** Results of IOS scores, environmental ratings, pro-environmental attitude, and carbon neutral behaviors in Study 3 (M ± SD)

Variables	Environmental self-identity	Environmentally friendly group (*n* = 60)	Environmentally unfriendly group (*n* = 60)	Environmentally neutral group (*n* = 60)
Environmental self-identity scores	High Environmental self-identity group (*n* = 30)	6.08 ± 0.53	5.80 ± 0.48	5.92 ± 0.49
	Low Environmental self-identity group (*n* = 30)	3.43 ± 0.85	3.59 ± 0.77	3.23 ± 0.78
Environmental ratings	High Environmental self-identity group (*n* = 30)	4.79 ± 1.53	2.02 ± 0.89	/
	Low Environmental self-identity group (*n* = 30)	4.55 ± 1.23	1.24 ± 0.87	/
Pro-environmental attitude	High Environmental self-identity group (*n* = 30)	5.47 ± 1.20	5.56 ± 1.26	5.54 ± 1.14
	Low Environmental self-identity group (*n* = 30)	4.06 ± 1.06	3.96 ± 1.25	5.02 ± 1.47
Carbon neutral behavior	High Environmental self-identity group (*n* = 30)	0.64 ± 0.15	0.67 ± 0.15	0.67 ± 0.16
	Low Environmental self-identity group (*n* = 30)	0.46 ± 0.18	0.47 ± 0.17	0.64 ± 0.17

### Pro-environmental attitude and carbon neutral behavior

Regarding pro-environmental attitude, the main effect of the group was significant, *F*_(2,174)_ = 3.43, *p* = 0.03, *η*_*p*_^*2*^ = 0.04; compared with the environmentally neutral behavior group (5.38 ± 1.24), those in the environmental friendly behavior group reported a lower pro-environmental attitude (4.84 ± 1.48, *t*_(118)_ = −2.18, *p* = 0.03); however, no significant difference was observed between the environmentally neural and unfriendly group (5.29 ± 1.24, *t*_(118)_ = −0.40, *p* = 0.69). The main effect of environmental self-identity was significant, *F*_(1, 174)_ = 11.82, *p* = 0.001, *η*_*p*_^*2*^ = 0.06; compared with the college students with low environmental self-identity (4.86 ± 1.41), those with high environmental self-identity reported a higher pro-environmental attitude (5.48 ± 1.19). Furthermore, their interaction was significant, *F* = 12.39, *p* < 0.001, *η*_*p*_^*2*^ = 0.13. Simple effect analysis revealed that for the college students with low environmental self-identity, pro-environmental attitude differed significantly among the three groups, *F*_(2, 175)_ = 10.09,* p* < 0.001; multiple comparisons revealed that compared with the neutral group (5.69 ± 1.12), both the environmentally friendly (4.10 ± 1.45, *t*_(58)_ = −4.77, *p* < 0.001) and unfriendly (4.78 ± 1.22, *t*_(58)_ = −3.02, *p* = 0.004) groups exhibited significantly less carbon neutral behavior, consistent with the *vicarious licensing and identity*; however, no significant differences were observed between the environmental friendly and unfriendly (*t*_(58)_ = −1.97, *p* = 0.06). For the college students with high environmental self-identity, no significant differences were observed among the three groups, *F*_(2, 175)_ = 0.32,* p* = 0.78 (environmentally friendly: 5.47 ± 1.20, unfriendly: 5.56 ± 1.26, neutral: 5.54 ± 1.14), *F*_(2, 175)_ = 0.30,* p* = 0.74 (see Table 3 and Fig. 2C).

For carbon neutral behavior, the main effect of group was significant, *F*_(2, 174)_ = 7.31,* p* = 0.001, *η*_*p*_^*2*^ = 0.08; multiple comparisons revealed that compared with the neutral group (0.66 ± 0.16), both the environmentally friendly (0.55 ± 0.19, *t*_(58)_ = −4.09, *p* < 0.001) and unfriendly groups (0.57 ± 0.19, *t*_(58)_ = −3.87, *p* < 0.001) exhibited significantly less carbon neutral behavior, indicating clear *vicarious licensing and identity*; however, no significant difference was observed between the environmental friendly and unfriendly behaviors group (*t*_(58)_ = −0.37, *p* = 0.71). The main effect of environmental self-identity was significant, *F*_(1, 174)_ = 28.80,* p* < 0.001, *η*_*p*_^*2*^ = 0.14: participants with high environmental self-identity selected more carbon neutral behavior (0.66 ± 0.15) than those with low environmental self-identity (0.53 ± 0.19). Their interaction was significant, *F*_(2, 174)_ = 4.76,* p* = 0.01, *η*_*p*_^*2*^ = 0.05. Simple effect analysis revealed that for the college students with low environmental self-identity, carbon neutral behavior differed significantly among the three groups, *F*_(2, 175)_ = 10.09,* p* < 0.001; multiple comparisons revealed that compared with the neutral group (0.64 ± 0.17), both the environmentally friendly (0.46 ± 0.18, *t*_(58)_ = −4.09, *p* < 0.001) and unfriendly groups (0.47 ± 0.17, *t*_(58)_ = −3.88, *p* < 0.001) exhibited significantly less carbon neutral behavior, consistent with the *vicarious licensing and identity*; however, no significant differences were observed between the environmental friendly and unfriendly groups (*t*_(58)_ = −0.37, *p* = 0.71). Regarding college students with high environmental self-identity, no significant differences were observed among the three groups (environmentally friendly: 0.64 ± 0.15, unfriendly: 0.67 ± 0.15, neutral: 0.67 ± 0.16), *F*_(2, 175)_ = 0.04,* p* = 0.91 (see Table 3 and Fig. 2C).

In addition, to rule out the possibility that high- and low-identity groups differed on other dimensions (e.g., gender, age, environmental knowledge), the two groups were compared on these variables. No significant differences emerged (*ps* > 0.20). When these variables were entered as covariates in the ANOVA, the critical interaction between group and environmental self-identity remained significant, *F*_(2, 170)_ = 4.52, *p* = 0.01, *η*_*p*_^*2*^ = 0.05, indicating that the moderating effect is specific to environmental self-identity rather than to other individual differences. To further scrutinize the robustness of the buffering effect, the carbon–neutral data were re-analyzed from Study 3 using an ANCOVA, with participants' baseline score on the Environmental Self-Identity scale (which was used for group selection) entered as a continuous covariate, alongside the categorical variable of high/low identity group. The critical interaction between the categorical environmental self-identity group and recall behavior remained significant, *F*_(2, 175)_ = 3.98, *p* = 0.02, *ηₚ*^*2*^ = 0.04. This suggests that the buffering effect is not merely an artifact of a ceiling effect but represents a genuine moderation by self-identity.

### Discussion of Study 3

Study 3 identified environmental self-identity as an internal buffer against the *vicarious trap*. Individuals with high environmental self-identity were unaffected by recalling a close friend's environmental behavior, whereas low-identity individuals remained susceptible. This extends prior work [17] by showing that a robust self-concept immunizes individuals against vicarious licensing and identification. However, the extreme-groups design, while increasing statistical power for detecting the interaction between identity and recall behavior, may inflate effect size estimates and limit generalizability to individuals with moderate identity levels. Future research should replicate these findings using continuous identity measures and representative samples.

## Study 4

### Methods

#### Participants

A total of 387 college students were screened with the Environmental Self-Identity Scale [44],3 items, alpha = 0.90) and the lowest 27% (scores < = 3.5) were selected to ensure a sample with initially low identity, allowing room for change. Sixty-six participants were randomly assigned to an activity group (*n* = 33) or a control group (*n* = 33). During the 30-day diary phase, five participants withdrew, leaving a final sample of 61 (intervention: *n* = 31; control: *n* = 30; 11 male; *M* age = 19.85, *SD* = 1.24). Given the limited sample size, Study 4 is best interpreted as exploratory. A post-hoc sensitivity analysis using G*Power 3.1 indicated that with *N* = 61, *α* = 0.05, and the observed effect size of *f* = 0.35 (corresponding to eta-squared = 0.11 for the three-way interaction), the achieved statistical power was 0.45. Therefore, results should be considered preliminary and warrant replication.

#### Design and procedure

A 3 (recall behavior: friendly, unfriendly, neutral) × 2 (group: activity, control) × 2 (time: pre‑test, post‑test) mixed design was used, with recall behavior and group as between‑subjects factors and time as a within‑subjects factor. Dependent variables were environmental self‑identity, pro‑environmental intentions, and carbon‑neutral behavior. Daily diary methods have been effectively employed to capture dynamic within-person processes in psychological research, including how daily states influence subsequent coping and well-being [30]. The procedure consisted of three phases:

- Pre‑test (Day 0): All participants completed the environmental self‑identity scale, pro‑environmental intentions measure, and carbon emission task (same as in Studies 1–3). They also nominated a close friend and completed a recall task for that friend’s environmental behavior (randomly assigned to friendly, unfriendly, or neutral condition). This provided a baseline measure of the vicarious trap.
- Activity session (Day 1): The activity group participated in a 90‑minute guided group session designed to enhance environmental self‑identity. The session integrated three components (see Table 4 for details): Cognitive: Presentation on the environmental impact of daily behaviors and the concept of “being a green person.” Affective: Viewing and discussing an emotionally evocative video about nature and human responsibility. Behavioral: A guided self‑reflection exercise (“My Green Self‑Portrait”) where participants listed their past pro‑environmental actions and envisioned their ideal green self, followed by a public commitment to engage in one new green behavior for the next 30 days. The control group engaged in a neutral discussion about campus life without environmental content.

**Table 4:** Outline of group counseling program for environmental self-identity

Unit Theme	Group Counseling Objective	Activity Content
We Are Together	Introduce the goals and nature of group counseling, sign the group commitment letter; Form a team, promote mutual understanding among members, and enhance familiarity; Form group norms and draw posters; Relax through meditation, end this activity	1. Snowball; 2. My self-Portrait; 3. My expectations; 4. Relaxation meditation
Hello, Environmental Protection	1. Stimulate group enthusiasm, introduce the concept of environmental protection; 2. Increase environmental knowledge reserves; 3. Through fun quizzes, further consolidate environmental knowledge	1. Throwing the unfaithful; 2. How much do you know about environmental protection; 3. Environmental knowledge competition; 4. Relaxation meditation
"Carbon" Aesthetically Perfect	1. Enliven the atmosphere, recognize the intricate connections in nature; 2. Trigger emotional resonance	1. Heartful knots; 2. Documentary viewing; 3. Relaxation meditation
Environmental Protection, Treasure of the Earth	1. Introduce the theme of garbage classification through fun games; 2. Further consolidate cognitive changes; 3. Immersive performance of environmental protection drama to stimulate environmental enthusiasm	1. Fun basketball; 2. How much do you know about garbage classification; 3. The birth of actors; 4. Relaxation meditation
I Am "Carbon" Passersby	1. Enliven the atmosphere, experience the negative consequences of destroying the environment; 2. Inspire daily environmental protection behaviors, enhance environmental self-identity; 3. Summarize the practical activities of "Environmental Guardian", share insights, guide college students to actively practice environmental protection	1. Self-Inflicted consequences; 2. Environmental checklist; 3. Environmental guardian; 4. Relaxation meditation
I Am Saying Goodbye with Fond Emotions	1. Enhance communication among members, enhance mutual feelings; 2. Strengthen the effect of group activities, generate transfer	1. Mindfulness meditation; 2. Recalling the past; 3. Sending good wishes; 4. Farewell

- Daily diary (Days 2–31): Starting the day after the activity session, all participants completed a brief online diary every evening between 21:30 and 23:00 for 30 days. Research assistants sent reminders at 23:00. Each diary took about 3 min and included: Daily environmental self‑identity (3 items adapted from [44],e.g., “Today, being environmentally friendly was an important part of who I am”; 1 = strongly disagree, 7 = strongly agree). Daily pro‑environmental intentions (2 items; e.g., “Today, I intended to act in an environmentally friendly way”; 1 = strongly disagree, 7 = strongly agree). Daily PEB frequency (checklist of 5 common behaviors: recycling, reducing energy use, using reusable bags, walking/biking instead of driving, avoiding single‑use plastics; sum of behaviors performed that day, range 0–5).
- Post‑test (Day 32): One day after the diary period, participants returned to the lab and completed the same measures as in the pre‑test (self‑identity, intentions, carbon emission task, and friend recall task). This allowed assessment of whether the activity’s effects persisted and whether the vicarious trap remained absent. In addition, all study materials and anonymized data, can be accessed via the following anonymized OSF link (https://osf.io/dtqyj/overview?view_only = 915fe15173cd4ce49d3ecc82356d3f96).

#### Data recording and analysis

First, a three-factor repeated measures analysis of variance (ANOVA) was conducted to examine the effects of the group-training intervention on environmental self-identity, pro-environmental intentions, and carbon–neutral behaviors.

Second, for the 30-day diary data, mixed-effects models with restricted maximum likelihood (REML) estimation were used to examine the temporal dynamics between daily environmental self-identity (R), pro-environmental intention (Y), and PEB frequency (T). Each model included autoregressive controls—the previous day's value of the dependent variable—to distinguish true cross-lagged effects from temporal stability. All Level-1 predictors were group-mean centered within each participant. Random intercepts and random slopes for the predictor were specified to account for between-person heterogeneity. Likelihood ratio tests evaluated the significance of random effects. Four cross-lagged paths were tested: Model 1 (Identity → Intention): Y_{t + 1} = β₀ + β₁R_t + β₂Y_t + ε; Model 2 (Intention → Identity): R_{t + 1} = β₀ + β₁Y_t + β₂R_t + ε; Model 3 (Identity → Behavior): T_{t + 1} = β₀ + β₁R_t + β₂T_t + ε; Model 4 (Behavior → Identity): R_{t + 1} = β₀ + β₁T_t + β₂R_t + ε. In each model, β₁ is the cross-lagged coefficient of primary interest, and β₂ is the autoregressive coefficient. Results are reported separately for the intervention (*n* = 31) and control (*n* = 30) groups. Complete fixed and random effects are presented in Table 6.

Third, a simple mediation model (Group → Post-test Identity → Carbon Behavior) was conducted using the PROCESS Model 4 logic [23] to test whether changes in environmental self-identity carried the intervention effect on carbon–neutral behavior. Post-test environmental self-identity (Day 30, controlling for Day 1) served as the mediator, and post-test carbon–neutral behavior (Day 30, controlling for Day 1) served as the outcome. As a sensitivity analysis, 30-day diary means were also used to increase measurement reliability. Bootstrap confidence intervals (5,000 resamples) tested the significance of indirect effects.

## Results

### Environmental self-identity and pro-environmental intentions

Regarding environmental self-identity, the main effect of the group was not significant (environmentally friendly: 4.05 ± 0.51, unfriendly: 3.74 ± 0.66, neutral: 3.84 ± 0.61), *F*_(2, 55)_ = 1.29,* p* = 0.29, *η*_*p*_^*2*^ = 0.05. However, the main effect of the intervention measure was significant, *F*_(1, 55)_ = 9.17,* p* = 0.004, *η*_*p*_^*2*^ = 0.14, indicating that the environmental self-identity level of the intervention group (4.10 ± 0.64) higher than that of the control group (3.64 ± 0.49). Furthermore, the main effect of the test was significant, *F*_(1, 55)_ = 78.97,* p* < 0.001, *η*_*p*_^*2*^ = 0.59, indicating that the participants reported a higher level of environmental self-identity in the post-test (4.38 ± 0.99) compared with that of the pre-test (3.37 ± 0.62). Their interaction effect was significant, *F*_(2, 55)_ = 38.25,* p* < 0.001, *η*_*p*_^*2*^ = 0.41; simple effect analysis revealed no significant difference in environmental self-identity between the two groups (intervention group: 3.25 ± 0.64; control group: 3.50 ± 0.57) in the pre-test, *F*_(1, 55)_ = 3.08,* p* = 0.09, *η*_*p*_^*2*^ = 0.05; however, the environmental self-identity of the intervention group (4.94 ± 1.00) was significantly higher than that of the control group (3.79 ± 0.55) in the post-test, *F*_(1, 55)_ = 29.17,* p* < 0.001, *η*_*p*_^*2*^ = 0.35. Simultaneously, no significant difference was observed in the environmental identity of the control group level between the pre-test (3.50 ± 0.57) and post-test (3.79 ± 0.55), *F*_(1, 55)_ = 3.19,* p* = 0.08, *η*_*p*_^*2*^ = 0.06; however, post-test level of the intervention group (4.94 ± 1.00) was significantly higher than that of the pre-test (3.25 ± 0.64), *F*_(1, 55)_ = 113.79,* p* < 0.001, *η*_*p*_^*2*^ = 0.67. Results revealed that the group counseling intervention significantly enhanced students’ environmental self-identity level (see Table 5).

**Table 5:** Results of environmental self-identity, pro-environmental attitudes, and carbon neutral behaviors in Study 4 (M ± SD)

Intervention measure	Group	Test	Environmental self-identity	Pro-environmental attitudes	carbon–neutral behavior
Intervention group (*n* = 31)	environmentally friendly group (*n* = 11)	Pre-test	3.33 ± 0.42	4.23 ± 0.61	0.52 ± 0.30
		Post-test	5.12 ± 1.01	5.44 ± 1.03	0.86 ± 0.28
	environmentally unfriendly group (*n* = 10)	Pre-test	3.34 ± 0.57	3.83 ± 1.42	0.51 ± 0.33
		Post-test	4.90 ± 0.90	5.02 ± 1.10	0.87 ± 0.17
	environmentally neutral group (*n* = 10)	Pre-test	3.07 ± 0.89	3.63 ± 1.60	0.80 ± 0.09
		Post-test	4.80 ± 1.13	5.30 ± 1.23	0.89 ± 0.18
Control group (*n* = 30)	environmentally friendly group (*n* = 11)	Pre-test	3.67 ± 0.58	4.37 ± 0.67	0.67 ± 0.29
		Post-test	4.00 ± 0.53	4.72 ± 0.64	0.62 ± 0.34
	environmentally unfriendly group (*n* = 9)	Pre-test	3.21 ± 0.60	4.15 ± 0.75	0.47 ± 0.30
		Post-test	3.58 ± 0.58	4.29 ± 0.45	0.42 ± 0.31
	environmentally neutral group (*n* = 10)	Pre-test	3.67 ± 0.42	4.12 ± 1.35	0.75 ± 0.09
		Post-test	3.83 ± 0.50	5.00 ± 1.20	0.79 ± 0.09

Regarding pro-environmental intentions, the main effects of the intervention measures were significant, *F*_(1, 55)_ = 4.49,* p* = 0.04, *η*_*p*_^*2*^ = 0.08, indicating that compared with the control group (5.18 ± 1.09), the intervention group had significantly higher pro-environmental intentions (4.62 ± 0.85). However, the main effect of the groups was not significant (environmentally friendly: 5.18 ± 1.07; unfriendly: 4.65 ± 0.98, neutral: 4.90 ± 0.96), *F*_(1, 55)_ = 1.27,* p* = 0.29, *η*_*p*_^*2*^ = 0.04. The main effect of the test was significant, *F*_(1, 55)_ = 9.91,* p* = 0.003, *η*_*p*_^*2*^ = 0.15, indicating that participants reported a higher level of pro-environmental intentions in the post-test (5.13 ± 1.06) compared with the pre-test (4.68 ± 1.31). Their interaction effect was significant, *F*_(1, 55)_ = 11.25,* p* = 0.001, *η*_*p*_^*2*^ = 0.17; simple effect analysis revealed no significant difference in pro-environmental intentions between the two groups (intervention group: 4.73 ± 1.53; control group: 4.63 ± 1.38) at the pre-test, *F*_(1, 55)_ = 0.02,* p* = 0.88, *η*_*p*_^*2*^ = 0.01; however, pro-environmental intentions of the intervention group (5.64 ± 0.88) in the post-test were significantly higher than those of the control group (4.61 ± 0.98), *F*_(1, 55)_ = 18.43,* p* < 0.001, *η*_*p*_^*2*^ = 0.25. Meanwhile, difference in pro-environmental intentions between the pre-test (4.63 ± 1.38) and post-test (4.61 ± 0.98) and was not significant in the control group, *F*_(1, 55)_ = 0.03,* p* = 0.87, *η*_*p*_^*2*^ = 0.01; however, the intervention group’s pro-environmental intentions in the post-test (5.64 ± 0.88) were significantly greater than those in the pre-test (4.73 ± 1.53), *F*_(1, 55)_ = 21.33,* p* < 0.001, *η*_*p*_^*2*^ = 0.28. This indicated that the group intervention significantly enhanced students’ pro-environmental intentions (see Table 5).

### Carbon neutral behavior

The main effect of the intervention measures was significant, *F*_(1, 55)_ = 5.15,* p* = 0.03, *η*_*p*_^*2*^ = 0.09; indicating that the intervention group (0.74 ± 0.19) made more carbon neutral behaviors compared with that of the control group (0.62 ± 0.27). Furthermore, the main effect of the group was significant, *F*_(2, 55)_ = 6.78,* p* = 0.002, *η*_*p*_^*2*^ = 0.20; indicating that the environmentally unfriendly behavior group made fewer carbon neutral behaviors (0.57 ± 0.26, *p* = 0.002) compared with the neutral behavior group (0.81 ± 0.11); however, no significant difference was observed between this group and the environmentally friendly behavior group (0.67 ± 0.25, *p* = 0.13). The main effect of the test was significant, *F*_(1, 55)_ = 14.48,* p* < 0.001, *η*_*p*_^*2*^ = 0.21, indicating that the participants made more carbon neutral behaviors in the post-test (0.74 ± 0.29) compared with that of the pre-test (0.62 ± 0.27). The third-interaction effect was significant, *F*_(2, 55)_ = 3.28,* p* = 0.04, *η*_*p*_^*2*^ = 0.11; simple effect analysis revealed that in the control group, significant differences were observed in carbon neutral behaviors among the three groups at the pre-test (*F*_(2, 55)_ = 3.45,* p* = 0.04, *η*_*p*_^*2*^ = 0.11) and post-test (*F*_(2, 55)_ = 6.08,* p* = 0.004, *η*_*p*_^*2*^ = 0.18), suggesting that the environmentally unfriendly behavior group (pre-test: 0.47 ± 0.30; post-test: 0.42 ± 0.31) made fewer carbon neutral behaviors compared with the neutral behavior group (pre-test: 0.75 ± 0.09; post-test: 0.79 ± 0.09, *ps* < 0.04); however, no significant difference was observed between this group and the environmentally friendly behavior group (pre-test: 0.67 ± 0.29; post-test: 0.62 ± 0.34, *ps* > 0.43). Simultaneously, significant differences were observed in carbon neutral behaviors among the three groups in the intervention group at pre-test (*F*_(2, 55)_ = 4.43,* p* = 0.02, *η*_*p*_^*2*^ = 0.14); multiple comparisons revealed that the carbon neutral behaviors of the environmentally friendly (0.52 ± 0.30) and unfriendly behavior groups (0.51 ± 0.33) were significantly less than that of the environmentally neutral behavior group (0.80 ± 0.09, *ps* < 0.04); however, the differences among the three groups were not significant at the post-test (environmentally friendly: 0.86 ± 0.29; unfriendly: 0.87 ± 0.17; neutral: 0.89 ± 0.18), *F*_(2, 55)_ = 0.03,* p* = 0.97, *η*_*p*_^*2*^ = 0.01 (see Table 5).

### Effect evaluation of diary method

**Fig. 3:**
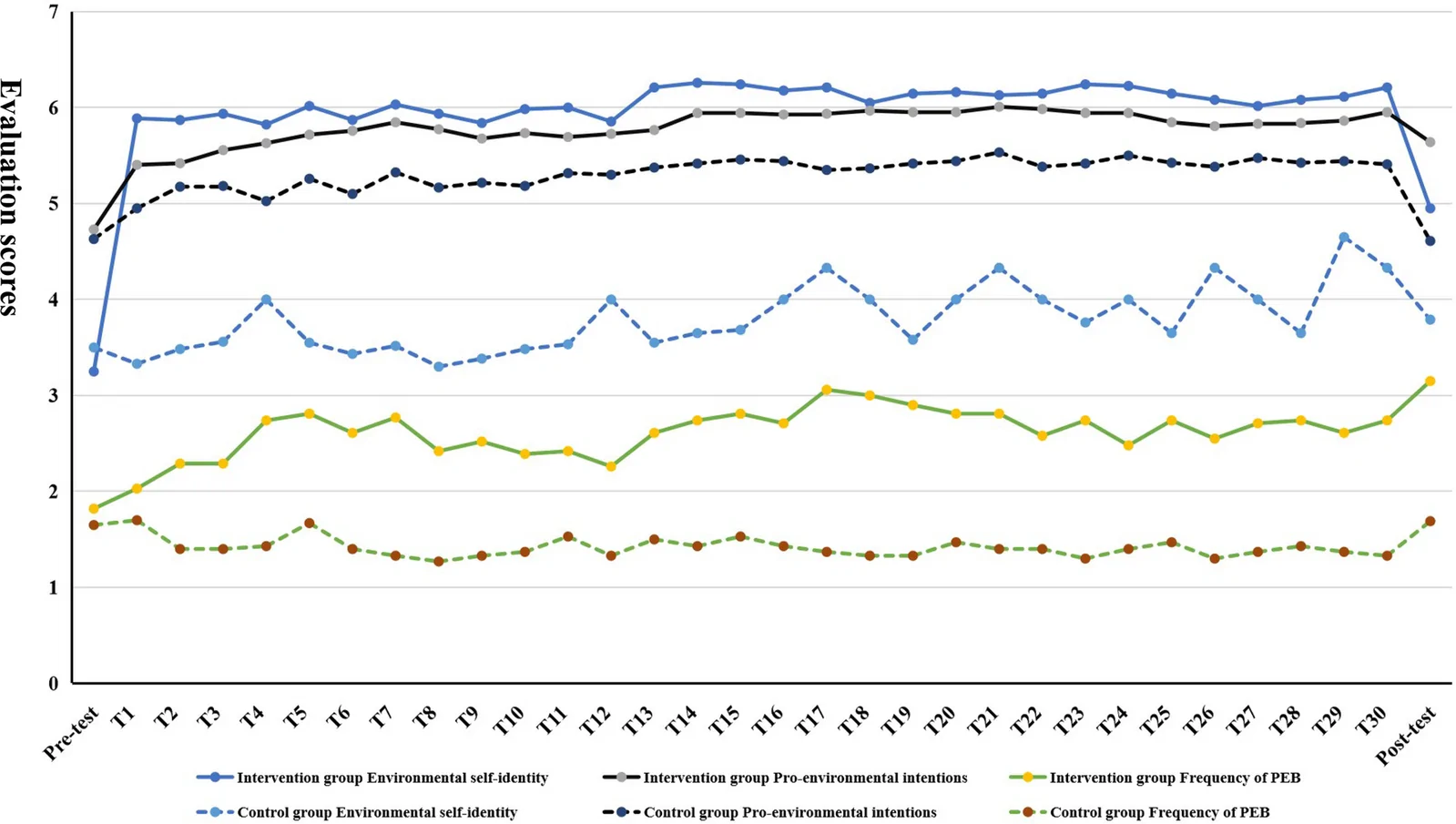
Diary method for tracking the daily changes in measurement indicators (environmental self-identity, pro-environmental intentions and frequency) between intervention and control group

Regarding the overall analysis, after the group counseling intervention, a 30-day diary-based tracking measurement was conducted on participants' environmental self-identity, pro-environmental intentions, and behavioral tendencies. Compared with the control group, the intervention group showed significant improvements in average daily environmental self-identity (*F*_(1, 59)_ = 8.28,* p* = 0.01, *η*_*p*_^*2*^ = 0.12), pro-environmental intentions (*F*_(1, 59)_ = 4.14, *p* = 0.05, *η*_*p*_^*2*^ = 0.07), and PEB frequency (*F*_(1, 59_) = 5.03, *p* < 0.001, *η*_*p*_^*2*^ = 0.37), maintaining relatively stable trends across 30 days. However, a decline in post-test scores was observed on the third day after the intervention, suggesting some attenuation over time (see Fig. 3).

### Cross-lagged analysis

Complete fixed and random effects are presented in Table 6. Regarding the cross relationship between pro-environmental intention and environmental self-identity, the forward path from identity to intention (Model 1) was not significant in either the intervention group *(β* = 0.04, *z* = 1.20, *p* = 0.23) or the control group (*β* = 0.01, *z* = 0.20, *p* = 0.83). In contrast, the reverse path from intention to identity (Model 2) was significant in both the intervention group (*β* = 0.45, *z* = 4.90, *p* < 0.001) and the control group (*β* = 0.26, *z* = 3.50, *p* < 0.001). This pattern indicates that pro-environmental intention functions as a robust antecedent of environmental self-identity in daily dynamics, regardless of intervention condition. Regarding the cross relationship between PEB frequency and environmental self-identity, the forward path from identity to behavior (Model 3) was significant in the intervention group (*β* = 0.22, *z* = 2.80, *p* = 0.01) but not in the control group (*β* = 0.01, *z* = 0.20, *p* = 0.84). The reverse path from behavior to identity (Model 4) was not significant in the intervention group (*β* = 0.06,* z* = 1.80, *p* = 0.07) and was significantly negative in the control group (*β* = − 0.13, *z* = − 2.90, *p* = 0.003), suggesting identity-behavior decoupling in the absence of intervention support. Random effects confirmed significant between-person variability in intercepts across all models (all *χ*^*2*^ > 430, *ps* < 0.001).

**Table 6:** Analysis table of lag effect in diary method

Path		fixed effect				random effect			
		**coefficient**	**error**	***t***	***p***	**standard deviation**	**variance component**	***χ***^***2***^	***p***
Environmental self-identity → Pro-environmental intentions									
Intervention group	intercept	5.83	0.15	38.73	< 0.001	0.85	0.72	4151.96	< 0.001
	slope	0.04	0.04	1.01	0.32	0.14	0.02	60.69	0.001
Control group	intercept	5.34	0.16	33.65	< 0.001	0.88	0.77	2402.60	< 0.001
	slope	0.01	0.03	0.24	0.81	0.09	0.01	34.32	0.19
Pro-environmental intentions → Environmental self-identity									
Intervention group	intercept	6.01	0.13	46.85	< 0.001	0.72	0.51	1128.09	< 0.001
	slope	−0.01	0.04	−0.16	0.87	0.12	0.01	36.68	0.13
Control group	intercept	5.49	0.13	43.72	< 0.001	0.69	0.47	904.44	< 0.001
	slope	−0.02	0.05	−0.50	0.62	0.16	0.02	44.13	0.03
Environmental self-identity → Frequency of PEB									
Intervention group	intercept	2.65	0.18	15.01	< 0.001	0.98	0.96	865.29	< 0.001
	slope	0.22	0.08	2.83	0.009	0.28	0.08	62.82	0.001
Control group	intercept	1.40	0.11	13.20	< 0.001	0.58	0.34	1188.65	< 0.001
	slope	0.01	0.02	0.06	0.95	0.09	0.01	24.44	0.50
Frequency of PEB → Environmental self-identity									
Intervention group	intercept	6.01	0.13	46.85	< 0.001	0.72	0.51	1215.17	< 0.001
	slope	0.13	0.08	2.00	0.06	0.32	0.10	163.56	< 0.001
Control group	intercept	4.49	0.13	43.72	< 0.001	0.69	0.47	729.49	< 0.001
	slope	0.08	0.06	1.45	0.16	0.24	0.06	87.14	< 0.001

However, given the modest sample size (*N* = 61), the non-significant paths—particularly identity to intention (Model 1) and behavior to identity (Model 4)—should be interpreted with caution, as limited statistical power may have prevented detection of smaller but theoretically meaningful effects. Future studies with larger samples are needed to confirm these null findings.

### Mediation analysis

We tested whether changes in environmental self-identity carried the intervention effect on carbon–neutral behavior (Group → Post-test environmental self-identity → PEB). The indirect effect was not significant using single-day pre-post measures (*ab* = 0.06, Bootstrap 95% CI [− 0.08, 0.31]). Using 30-day diary means for greater measurement reliability, the indirect effect was significant: *ab* = 0.39, 95% CI [0.09, 0.78] (*a* = 0.50, *p* = 0.01; *b* = 0.78, *p* < 0.001). This indicates that the intervention promoted carbon–neutral behavior by enhancing environmental self-identity (see Table 7).

**Table 7:** Mediation analysis among group, environmental self-identity and PEB

Model	Path	*b*	*SE*	*t/z*	*p*	*95% CI*
Model A: Single-day pre-post (Day 1 & Day 30, controlling baseline)	c (total effect): Group → Post CB	0.97	0.25	3.85	< 0.001	—
	a: Group → Post ESI (R30)	0.58	0.23	2.49	0.02	—
	b: Post ESI (R30) → Post CB (M30)	0.11	0.15	0.73	0.47	—
	c' (direct): Group → Post CB	0.88	0.27	3.21	0.002	—
	ab (indirect effect)	0.06	—	0.70	0.48	[− 0.08, 0.31]
Model B: 30-day means (sensitivity analysis)	a: Group → Mean ESI (MR)	0.50	0.19	2.63	0.01	—
	b: Mean ESI (MR) → Mean CB (MD)	0.78	0.17	4.51	< 0.001	—
	c' (direct): Group → Mean CB (MD)	− 0.98	0.27	− 3.64	< 0.001	—
	ab (indirect effect)	0.39	—	2.27	0.02	[0.08, 0.78]

*ESI* Environmental Self-Identity, *CB* Carbon–neutral Behavior, *MR* Mean ESI (30-day diary), *MD* Mean Carbon–neutral Behavior. Model A controls for baseline (Day 1) values of the mediator (R1) and dependent variable (M1). Model B uses 30-day diary means for greater measurement reliability. Indirect effects tested with Bootstrap 95% confidence intervals (5,000 resamples). CI excluding zero indicates significant mediation

Additionally, given the exploratory nature of Study 4 and the limited sample size, we report effect sizes alongside *p*-values and focus on patterns of results. The three-way interaction (Group × Recall Behavior × Time) for carbon–neutral behavior yielded *F*_(2, 55)_ = 3.28, *p* = 0.04, *η*_*p*_^*2*^ = 0.11. However, post-hoc power was low (estimated power = 0.45). Therefore, these findings should be considered preliminary and warrant replication.

### Discussion of Study 4

Study 4 provided preliminary evidence that environmental self-identity is malleable and causally protective. With autoregressive controls, identity significantly predicted next-day PEB in the intervention group, whereas the reverse path was not significant. This asymmetry suggests that the identity-to-behavior pathway requires activation—here provided by the group activity—and that behavioral feedback alone is insufficient to spontaneously reinforce self-concept. Mediation analyses confirmed identity enhancement as the causal mechanism. However, these findings are exploratory given the modest sample size and should be interpreted as hypothesis-generating for future pre-registered studies.

## General discussion

Across four studies, evidence was found for a vicarious trap in pro-environmental behavior: close others' good deeds license personal inaction, and their bad deeds justify it. Two effective buffers were identified—external situational openness and internal environmental self-identity—and initial evidence was provided that self-identity can be cultivated to initiate an identity-driven behavioral pathway.

### The asymmetric nature of vicarious moral regulation in the environmental domain

A central finding across all studies is the pronounced asymmetry in vicarious moral regulation. While both vicarious licensing and identification reliably reduced participants' PEB, vicarious cleansing—the compensatory increase in moral behavior after observing others' transgressions—was entirely absent. This asymmetry may stem from the unique characteristics of environmental harm: its diffuse, temporally distant, and victimless nature facilitates moral disengagement and suppresses the motivation to compensate [15, 28]. Unlike interpersonal transgressions that involve identifiable victims and evoke guilt and empathy, environmental harm lacks the emotional triggers necessary for cleansing. Consequently, the moral scale tips easily toward slackening but resists self-correction. This challenges the core assumption of moral balancing theory that individuals are motivated to maintain a stable moral self-image through compensatory actions [38], and underscores the necessity of external and internal buffers to counteract this built-in asymmetry.

### The role of psychological closeness and developmental context: for whom does the trap exist?

Study 1 demonstrated that the vicarious trap operates through psychological closeness rather than kinship: recalling loved ones' environmental behaviors triggered vicarious licensing and identification, whereas recalling family members' behaviors did not. This underscores that vicarious moral regulation is fundamentally a function of self-other overlap [1]. The IOS data confirmed greater overlap with loved ones than with family members, consistent with prior work showing that psychological closeness—not mere group membership—enables vicarious effects [18], [25].

The null family effect likely reflects developmental specificity. A meta-analysis by Duarte et al. [13] demonstrated that family influence on environmental attitudes declines significantly during emerging adulthood, when peer influence becomes dominant. Deng et al. [12] showed that children, but not young adults, effectively transmit pro-environmental norms to parents, suggesting a developmental asymmetry in family-based environmental socialization. During emerging adulthood, the primary developmental task shifts toward achieving intimacy with peers and romantic partners [39], and peers become the primary reference group for norm formation and identity exploration [7]. The vicarious trap is thus selectively activated by those with whom individuals share the greatest psychological overlap—for emerging adults, peers and romantic partners rather than family members. Identifying this boundary condition is crucial for both theoretical precision and intervention targeting: environmental programs for younger adolescents may benefit from family involvement, whereas programs for emerging adults should leverage peer networks.

### Breaking the trap from the outside: situational openness as an external buffer

Study 2 demonstrated that making the recalled behavior public eliminated the vicarious trap, even though participants' own choices remained private. An integrated two-process model is proposed. First, the situational openness transforms the recalled behavior from a private memory into a social fact, activating descriptive and injunctive norms [9]. When participants learn that a friend's environmental behavior will be disclosed to others, the behavior becomes a matter of public record, making the social implications of that behavior salient. Second, the situational openness engages impression management motives [22], as participants anticipate that their own environmental decisions may be evaluated against the backdrop of their friend's publicized behavior. The mechanism is more accurately characterized as a shift in normative inference rather than personal accountability: publicizing a friend's behavior signals what is considered socially appropriate, thereby overriding private moral rationalizations. This external buffer operates through social-normative pathways, suggesting its effectiveness may be strongest when behavior is visible to valued reference groups and when the publicized behavior carries clear normative implications.

### Environmental self-identity as an internal anchor and driver

Whereas situational openness operates through external social pressures, Studies 3 and 4 elucidated an internal resource that confers immunity to the vicarious trap and can be cultivated to drive sustained behavioral change.

Study 3 demonstrated that high environmental self-identity provides complete immunity to the trap: individuals who strongly identified as environmentally friendly were unaffected by recalling a friend's behavior. According to self-concept theory, identity-central values guide behavior through stable internal standards rather than situational cues [47]. This buffering effect held when controlling for baseline attitudes, ruling out the alternative explanation of stronger pre-existing attitudes [17]. Meta-analytic evidence confirms that environmental self-identity is among the strongest predictors of PEB, with effects persisting beyond those of attitudes and norms [42].

Study 4 extended these findings in two critical ways. First, it demonstrated that identity is malleable: a brief group activity integrating cognitive, affective, and self-reflective components increased self-identity and eliminated the trap at one-month follow-up. Mediation analyses confirmed identity enhancement as the causal mechanism, providing experimental evidence that extends the value-identity-behavior model [43]. Second, the 30-day diary data, analyzed with rigorous autoregressive controls, revealed the dynamic operation of identity in daily life. Identity significantly predicted next-day PEB in the intervention group but not in controls, indicating that the identity-to-behavior link requires activation rather than operating automatically. This is consistent with identity-based motivation theory, which holds that identities guide behavior only when cognitively accessible in the decision context [34]. The reverse path from behavior to identity was not significant in the intervention group and was significantly negative in controls, suggesting that daily PEB alone does not automatically strengthen identity—consistent with self-perception theory's prediction that self-inference requires perceived autonomy [3]. The negative path in controls further suggests that without intervention, engaging in PEB may feel externally motivated, paradoxically weakening identity—a pattern reminiscent of the overjustification effect [11].

Intention significantly predicted next-day identity but not next-day behavior, revealing a directional flow—intention to identity to behavior—that positions self-identity as a bridge across the intention-behavior gap [41]. Intentions that remain at the level of abstract preferences are easily overridden by situational pressures such as the vicarious trap; intentions that become part of identity gain motivational force because acting on them affirms who one is [34]. This identity-as-bridge model is consistent with longitudinal evidence that self-identity mediates the intention-behavior relationship over time [8], and with diary studies showing that identity-behavior associations are strongest when identity is frequently activated in daily life [26]. These findings suggest that interventions aiming to promote sustained PEB should target identity internalization—helping individuals become someone who cares—rather than merely boosting intentions.

### General theoretical integration: toward an open dual-system framework

The dual-pathway framework [49] posits that vicarious moral regulation operates through moral self-regulation and vicarious regulation, both converging to reduce PEB through self-other overlap [1]. Critically, this framework is characterized by profound asymmetry: vicarious cleansing is rarely observed in the environmental domain, as replicated across all four studies in the present research. The present findings add a third, countervailing pathway: identity-driven behavioral activation. When environmental self-identity is strengthened, it shifts the reference point for behavioral decisions from 'what have others done?' (vicarious regulation) and 'what have I done before?' (self-regulation) to 'who am I?' (identity-based regulation). This extension transforms the dual-system framework from a descriptive model—documenting how moral regulation operates—into a prescriptive one—identifying how its negative effects can be counteracted.

This identity-driven pathway aligns with Self-Determination Theory [37]: environmental self-identity reflects integrated regulation—the most autonomous form of extrinsic motivation in which behaviors are fully assimilated into the self. Identity-based interventions facilitate the internalization process from external regulation ('I should recycle because others expect it') to integrated regulation ('I recycle because I am an environmentally friendly person'). The identity-as-bridge model (intention to identity to behavior) further suggests that the intention-behavior gap exists because intentions must be internalized into the self-concept before translating into consistent action [8, 34]. Longitudinal research in health behavior has similarly demonstrated that self-identity mediates the intention-behavior relationship over time, supporting the generalizability of this mechanism across behavioral domains. Meta-analytic evidence confirms that environmental self-identity is a stronger predictor of PEB than social norms or perceived behavioral control [42, 46], supporting the primacy of the identity-driven pathway in promoting sustained behavioral change.

Regarding practical implications, the buffering effect of situational openness suggests that making environmental behavior visible—through public pledges, community challenges, or sustainability dashboards—can leverage social norms to neutralize the vicarious trap. The identity-driven pathway suggests that effective interventions should include active self-reflection components (e.g., 'My Green Self-Portrait'), provide opportunities for identity-congruent action soon after the intervention, and incorporate periodic booster sessions given the observed post-intervention decline. The 90-min group activity tested in Study 4 provides a scalable, low-cost model for educational and organizational settings. For emerging adults, peer-based programs may be more effective than family-based approaches. The finding that intention alone does not drive behavior suggests that public campaigns should target identity internalization rather than merely boosting intentions—a shift from convincing people to care toward helping people become someone who cares [45]. Regarding the null effect for family members, environmental education programs targeting younger adolescents—when family influence on environmental attitudes is stronger [13]—may be more effective than programs targeting emerging adults, whose environmental attitudes are more strongly shaped by peers.

### Limitations and future directions

Several limitations should be acknowledged, each suggesting clear paths for future inquiry. First, the modest sample size in Study 4 (*N* = 61) yielded low post-hoc power (0.45) for the three-way interaction, rendering the cross-lagged and mediation results preliminary. The non-significant paths—identity to intention (Model 1) and behavior to identity (Model 4)—warrant particular caution, as limited power may have precluded detection of smaller yet theoretically meaningful effects. Future pre-registered studies with larger samples and more frequent measurement waves are needed to confirm these exploratory patterns. Second, the culturally homogeneous Chinese student sample limits generalizability to non-WEIRD or older populations, and the null family effect may be specific to emerging adulthood rather than universal. Cross-cultural and developmental replications—comparing adolescents, emerging adults, and middle-aged groups—would clarify whether the vicarious trap and its buffers shift with life-stage reference groups. Specifically, environmental education programs targeting younger adolescents—when family influence on environmental attitudes remains strong [13]—may benefit from family involvement, whereas programs for emerging adults should capitalize on peer networks. Third, the diary method relied exclusively on self-reports, rendering the findings susceptible to common-method variance and social desirability biases. Future designs incorporating behavioral traces (e.g., digital footprints, peer nominations) or implicit measures would enhance validity and reduce subjective bias. Fourth, the extreme-groups design in Study 3 may inflate effect sizes relative to continuous-variable approaches and limits generalizability to individuals with moderate identity levels. Future research should treat environmental self-identity as a continuous predictor and replicate the moderation using regression-based frameworks. Fifth, the self-recall condition in Study 1, included as a benchmark, may reflect domain-general priming rather than vicarious processes, as it does not operate through self-other overlap. Subsequent studies should disentangle these mechanisms using experimental manipulations that isolate vicarious inference from mere concept accessibility. Sixth, the null family effect remains ambiguous because Study 1 did not differentiate parents, siblings, or other relatives. Collecting detailed relational data (e.g., closeness, interaction frequency, role) and employing dyadic designs would clarify whether kinship or psychological closeness underlies the effect. Finally, although Study 4 revealed an identity-driven forward pathway, the reverse pathway—behavior strengthening identity—was not significant, possibly due to insufficient power or reflecting a genuine asymmetry. Longer-term intensive longitudinal designs with adequate power and embedded behavioral nudges are necessary to test whether reciprocal reinforcement emerges under optimal conditions. Addressing these limitations in future work would not only strengthen causal inferences but also refine the dual-buffering framework and inform tailored interventions across diverse populations and developmental contexts.

## Conclusion

In conclusion, this research reveals a *vicarious trap* in pro-environmental behavior and identifies two buffers: situational openness and environmental self-identity. Study 4 demonstrated that self-identity is malleable—a brief group activity enhanced identity and eliminated the trap at one-month follow-up. Daily diary data with autoregressive controls revealed an identity-driven behavioral pathway, and mediation analyses confirmed identity enhancement as the causal mechanism. These findings suggest that interventions cultivating environmental self-identity—by helping individuals internalize pro-environmental values into their self-concept—may create self-sustaining behavioral change that resists the vicarious traps embedded in close relationships.

## Supplementary Information

Supplementary Material 1.

## Data Availability

All study materials, anonymized data, analysis code, and codebook can be accessed via the following anonymized OSF link (https://osf.io/dtqyj/overview?view_only=915fe15173cd4ce49d3ecc82356d3f96).
