# Supplementary material for: Breaking the vicarious trap: two buffers against close others’ environmental influence
Source: BMC Psychol. 2026 Oct 2;14:1449. doi: 10.1186/s40359-026-05363-y (PMC13644283; doi:10.1186/s40359-026-05363-y)
Supplement: Supplementary file 1 [file 40359_2026_5363_MOESM1_ESM.zip › materials and data/materials/Study 1/1回忆想象任务-控制组.pdf]

亲爱的同学：

您好！非常感谢您抽取宝贵的时间来参与本次实验研究，您填写的每一个答案对我们来说都十分重要，希望您认真作答。本研究所得数据仅供科学研究之用并严格保密，请您放心作答，感谢您的支持与帮助！

个人情况

年龄：　　　　　　　　　　　　　　　　性别：

恋爱时长：（　　）个月

【自我版】

问卷 1

首先，请阅读下列在现实生活中可能遇到的一个情境，仔细阅读这个情境中发生的故事，尽可能想象你自己是该情境中的主人公，当这个情境在你脑海中比较清楚时，请你按要求完成后面的问题。

情境 1：我和朋友们约好假期要出去游玩，大家意见不一致，有说去爬山的，有说去逛街的，还有说去密室逃脱的，而我更想要去郊外野炊。一番协调下，我们终于决定去爬山和野炊了。

请问：你们最后去了哪里？

____

____

____

____

____

____

情境 2：我是一个篮球爱好者，最近喜欢上了一个新晋篮球明星。我和室友聊天发现，他/她也和我一样，我感到非常惊喜。于是我们相约一起追星，一起打篮球，成为了很好的朋友。

请问：<u>你和室友因为什么成为了好朋友？</u>

____

____

____

____

问卷 2

请您根据上面两个情境，再思考与回忆一下你**自己**之前还有做过**类似行为**的经历吗？请花几分钟时间回忆与组织语言，并用笔写下一次**你自己做过的、让你最难忘的类似行为的事情**，要求不少于 100 字。

____

____

____

____

____

____

____

____

请问：<u>你过去的行为对你现在有什么影响？</u>

____

____

____

问卷 3

请您根据自己的真实感受与意愿，对下列 6 个条目的内容进行主观意愿的等级评定（打“√”），其中 1 代表完全不同意，7 代表完全同意。该量表的结果评定无好坏之分，只需要您真实反应填写即可。

项 目	1=完全不同意 —— 7=完全同意					
1. 我愿意签署一份请愿书来支持环保事业。	1	2	3	4	5	6	7
2. 我会考虑加入一个环保团体或俱乐部。	1	2	3	4	5	6	7
3. 我愿意缴纳更多的个人所得税来支持政府用于环境治理。	1	2	3	4	5	6	7
4. 如果能够让空气变得更加洁净，我愿意支付更多的电费。	1	2	3	4	5	6	7
5. 如果我经常购物的公司涉嫌环境污染，我将不再光顾，即使这让我的生活变得不那么方便。	1	2	3	4	5	6	7
6. 我愿意做出个人牺牲来减少环境污染，即使见效不那么快。	1	2	3	4	5	6	7

【亲人版】

1、请你集中精神想像特定的一个**亲人**____________（提供称谓：如爸爸、妈妈、叔叔、姑姑、姨妈、哥哥等）作为本次实验的目标人物，即**一个你最亲密的亲人，你们无话不谈，关系非常要好，是对你来说一个非常非常重要的人。**

2、下图为 7 对重叠程度不同的圆圈，一个代表**自己**，另一个代表你的**亲人**，交叉部分代表的是自我和他人重叠的程度（自我-他人重叠是指交往关系中的个体不同程度地接受和理解他人的观念、资源，从而出现自我和他人信息表征的重叠现象）。请在下面的图形中选择最符合**你和亲人**关系的图形（打“√”）。

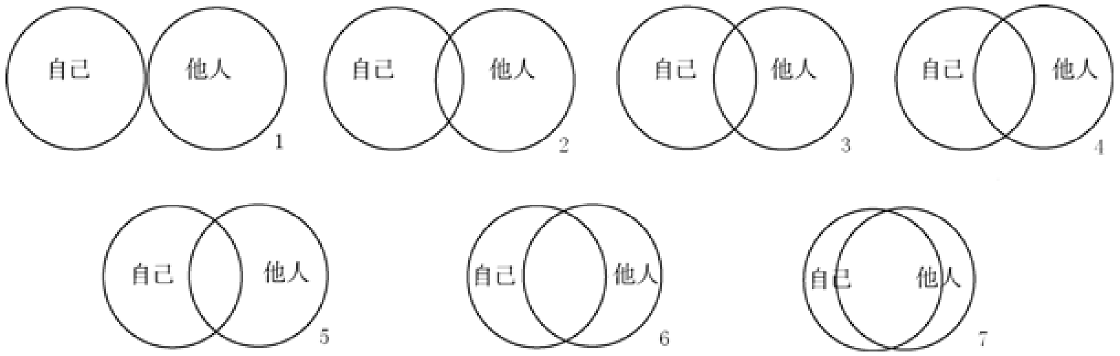

问卷 1

首先，请阅读下列在现实生活中可能遇到的一个情境，仔细阅读这个情境中发生的故事，尽可能想象你的**亲人**是该情境中的主人公，当这个情境在你脑海中比较清楚时，请你按要求完成后面的问题。

情境 1：________（亲人称谓）热衷于跳舞，空闲时间经常约着朋友们一起娱乐放松。________（亲人称谓）还组建了一个舞蹈团队，不时地出去参加一些演出，生活过得有滋有味。

请问：他/她喜欢做什么？

____

____

____

____

情境 2：_________（亲人称谓）与一帮好友约定出去旅游，对于去哪里大家意见不一。有想去周边游的，有想去海边玩的，还有想去江南小镇的，而________（亲人称谓）一如既往地等待大家做决定。最后大家决定去周边游玩。

请问：他/她们最后去了哪里？

____

____

____

____

____

问卷 2

请您根据上面两个情境，再思考与回忆一下你的亲人之前还有做过**类似行为**的经历吗？请花几分钟时间回忆与组织语言，并用笔写下一次**亲人做过的、让你最难忘的类似行为的事情（与你无关的）**，要求不少于 100 字。

请问：<u>他/她的行为对你有什么影响？</u>

问卷 3

请您根据自己的真实感受与意愿，对下列 6 个条目的内容进行主观意愿的等级评定（打“√”），其中 1 代表完全不同意，7 代表完全同意。该量表的结果评定无好坏之分，只需要您真实反应填写即可。

项　目	1=完全不同意 ———— 7=完全同意						
1. 我愿意签署一份请愿书来支持环保事业。	**1**	**2**	**3**	**4**	**5**	**6**	**7**
2. 我会考虑加入一个环保团体或俱乐部。	**1**	**2**	**3**	**4**	**5**	**6**	**7**
3. 我愿意缴纳更多的个人所得税来支持政府用于环境治理。	**1**	**2**	**3**	**4**	**5**	**6**	**7**
4. 如果能够让空气变得更加洁净，我愿意支付更多的电费。	**1**	**2**	**3**	**4**	**5**	**6**	**7**
5. 如果我经常购物的公司涉嫌环境污染，我将不再光顾，即使这让我的生活变得不那么方便。	**1**	**2**	**3**	**4**	**5**	**6**	**7**
6. 我愿意做出个人牺牲来减少环境污染，即使见效不那么快。	**1**	**2**	**3**	**4**	**5**	**6**	**7**

【恋人版】

1、请你集中精神想像您的**恋人**____________（提供昵称）作为本次实验的目标人物。

2、下图为 7 对重叠程度不同的圆圈，一个代表**自己**，另一个代表你的**恋人**，交叉部分代表的是自我和他人重叠的程度（自我-他人重叠是指交往关系中的个体不同程度地接受和理解他人的观念、资源，从而出现自我和他人信息表征的重叠现象）。请在下面的图形中选择最符合**你和恋人**关系的图形（打“√”）。

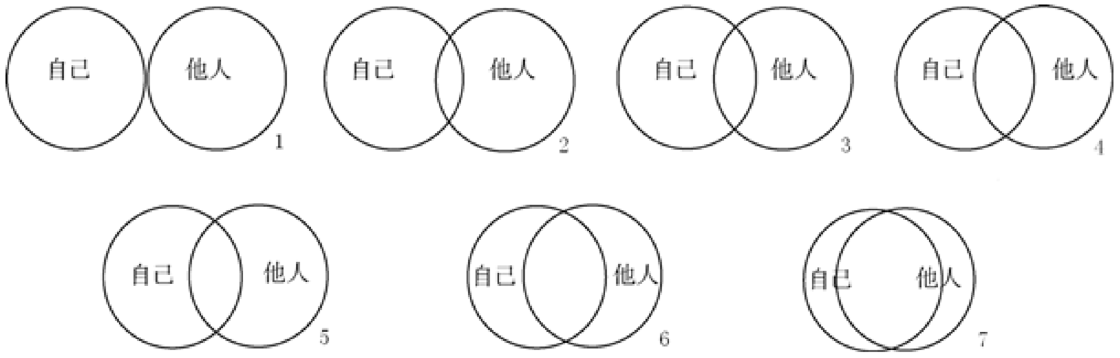

问卷 1

首先，请阅读下列在现实生活中可能遇到的一个情境，仔细阅读这个情境中发生的故事，尽可能想象你亲密的**恋人**是该情境中的主人公，当这个情境在你脑海中比较清楚时，请你按要求完成后面的问题。

情境 1：________（恋人昵称）和朋友们约好假期要出去游玩，大家意见不一致，有说去爬山的，有说去逛街的，还有说去密室逃脱的，而__________（恋人昵称）更想要去郊外野炊。一番协调下，他/她们终于决定去爬山和野炊了。

请问：他/她们最后去了哪里？

____

____

____

____

情境 2：_________（恋人昵称）是一个篮球爱好者，最近喜欢上了一个新晋篮球明星。________（恋人昵称）和室友聊天发现，他/她们喜欢的球星是同一个人，________（恋人昵称）感到非常惊喜。于是他/她们相约一起追星，一起打篮球，成为了很好的朋友。

请问：他/她们因为什么成为了好朋友？

____

____

____

____

____

问卷 2

请您根据上面两个情境，再思考与回忆一下你的**恋人**之前还有做过**类似行为**的经历吗？请花几分钟时间回忆与组织语言，并用笔写下一次**恋人做过的、让你最难忘的类似行为的事情（与你无关的）**，要求不少于 100 字。

请问：<u>他/她的行为对你有什么影响？</u>

问卷 3

请您根据自己的真实感受与意愿，对下列 6 个条目的内容进行主观意愿的等级评定（打“√”），其中 1 代表完全不同意，7 代表完全同意。该量表的结果评定无好坏之分，只需要您真实反应填写即可。

项 目	**1=完全不同意 —————— 7=完全同意**						
1. 我愿意签署一份请愿书来支持环保事业。	**1**	**2**	**3**	**4**	**5**	**6**	**7**
2. 我会考虑加入一个环保团体或俱乐部。	**1**	**2**	**3**	**4**	**5**	**6**	**7**
3. 我愿意缴纳更多的个人所得税来支持政府用于环境治理。	**1**	**2**	**3**	**4**	**5**	**6**	**7**
4. 如果能够让空气变得更加洁净，我愿意支付更多的电费。	**1**	**2**	**3**	**4**	**5**	**6**	**7**
5. 如果我经常购物的公司涉嫌环境污染，我将不再光顾，即使这让我的生活变得不那么方便。	**1**	**2**	**3**	**4**	**5**	**6**	**7**
6. 我愿意做出个人牺牲来减少环境污染，即使见效不那么快。	**1**	**2**	**3**	**4**	**5**	**6**	**7**

问卷 4（RISC）

请您根据自己的真实感受与意愿，对下列 9 个条目的内容进行主观意愿的等级评定（打“√”），其中 1 代表完全不同意，7 代表完全同意。该量表的结果评定无好坏之分，只需要您真实反应填写即可。

1=完全不同意 2=不同意 3=有点不同意 4=不确定 5=有点同意 6=同意 7=完全同意

此处的亲近关系指与亲近他人的关系，即与父母、朋友、恋人等的关系。

**题　目**	**1=完全不同意 ——————— 7=完全同意**						
1. 我的亲近关系是“我是什么样的人”的重要反映。	**1**	**2**	**3**	**4**	**5**	**6**	**7**
2. 当我和某人很亲近时，我感觉他是“我”的重要组成部分。	**1**	**2**	**3**	**4**	**5**	**6**	**7**
3. 我常常为我朋友的重大成就而自豪。	**1**	**2**	**3**	**4**	**5**	**6**	**7**
4. 我认为通过观察和了解我的好朋友可以获得我的某些重要特征。	**1**	**2**	**3**	**4**	**5**	**6**	**7**
5. 当我审视自己时，我通常会同时想起我的密友或家人。	**1**	**2**	**3**	**4**	**5**	**6**	**7**
6. 如果有人伤害了我亲近的人，我会感觉自己也受到了伤害。	**1**	**2**	**3**	**4**	**5**	**6**	**7**
7. 一般来说，我的亲近关系是自我形象的重要组成部分。	**1**	**2**	**3**	**4**	**5**	**6**	**7**
8. 我的自豪感来自于我了解我的挚友。	**1**	**2**	**3**	**4**	**5**	**6**	**7**
9. 当我与某人建立亲近关系时，我通常会非常认同这个人。	**1**	**2**	**3**	**4**	**5**	**6**	**7**

问卷 5

请您根据自己的真实感受与意愿，对下列 3 个条目的内容进行主观意愿的等级评定，其中 1 代表完全不同意，7 代表完全同意。该量表的结果评定无好坏之分，只需要您真实反应填写即可。

1=完全不同意 2=不同意 3=有点不同意 4=不确定 5=有点同意 6=同意 7=完全同意

题 目	1=完全不同意 ———————— 7=完全同意						
1. 凡事注重环保是我个人的重要特质。	**1**	**2**	**3**	**4**	**5**	**6**	**7**
2. 我属于一举一动都很注重环保的人。	**1**	**2**	**3**	**4**	**5**	**6**	**7**
3. 我认为自己是一个环保的人。	**1**	**2**	**3**	**4**	**5**	**6**	**7**
