# Supplementary material for: Breaking the vicarious trap: two buffers against close others’ environmental influence
Source: BMC Psychol. 2026 Oct 2;14:1449. doi: 10.1186/s40359-026-05363-y (PMC13644283; doi:10.1186/s40359-026-05363-y)
Supplement: Supplementary file 1 [file 40359_2026_5363_MOESM1_ESM.zip › materials and data/materials/Study 1/1回忆想象任务-环保不友好组.pdf]

亲爱的同学：

您好！非常感谢您抽取宝贵的时间来参与本次实验研究，您填写的每一个答案对我们来说都十分重要，希望您认真作答。本研究所得数据仅供科学研究之用并严格保密，请您放心作答，感谢您的支持与帮助！

个人情况

年龄：　　　　　　　　　　　　　　　　　性别：

恋爱时长：（　　）个月

【自我版】

问卷 1

首先，请阅读下列在现实生活中可能遇到的一个情境，仔细阅读这个情境中发生的故事，尽可能想象你**自己**是该情境中的主人公，当这个情境在你脑海中比较清楚时，请你按要求完成后面的问题。

情境 1：我家里有很多垃圾需要丢弃，现在需要将其分离并带到垃圾收集点。我不耐烦地把不同种类的垃圾分开，并把它们都扔在一个大袋子里。然后，我走到垃圾收集站，看到剩余的垃圾桶已经满了，就把装着各种垃圾的大袋子扔进了厨余垃圾桶里。

请问：（1）你做了什么？

____

____

____

（2）该行为属于环境不友好的行为吗？（1 = 是，2 = 否,打√）。

（3）请评估该行为的环保程度（1 = 极其不环保，2 = 有点不环保；3 = 不确定；4 = 非常环保；5 = 绝对环保；打√）。

情境 2：学校第五教学楼附近有一个湖泊，来来往往的学生很多，我刚好在附近有美术课，每次上完课同学们都争抢着去洗手间清洗用具，因为嫌排队太麻烦，我好几次将美术课未用完的颜料墨水倒进湖中，路过的同学指指点点，我依然我行我素，不为所动。

请问：（1）<u>你做了什么？</u>

____

____

____

（2）该行为属于环境不友好的行为吗？（1 = 是，2 = 否,打√）。

（3）请评估该行为的环保程度（1 = 极其不环保，2 = 有点不环保；3 = 不确定；4 = 非常环保；5 = 绝对环保；打√）。

问卷 2

请您根据上面两个情境，再思考与回忆一下你**自己**之前还有做过类似的**环境不友好行为**的经历吗？请花几分钟时间回忆与组织语言，并用笔写下一次**你做过的、让你最难忘的环境不友好行为的事情**，要求不少于 100 字。

____

____

____

____

____

____

____

____

____

请问：（1）<u>你做了什么？</u>

____

____

____

（2）该行为属于环境不友好的行为吗？（1 = 是，2 = 否,打√）。

（3）请评估该行为的环保程度（1 = 极其不环保，2 = 有点不环保；3 = 不确定；4 = 非常环保；5 = 绝对环保；打√）。

问卷 3

请您根据自己的真实感受与意愿，对下列 6 个条目的内容进行主观意愿的等级评定（打“√”），其中 1 代表完全不同意，7 代表完全同意。该量表的结果评定无好坏之分，只需要您真实反应填写即可。

项　目	1=完全不同意 ———— 7=完全同意						
1. 我愿意签署一份请愿书来支持环保事业。	**1**	**2**	**3**	**4**	**5**	**6**	**7**
2. 我会考虑加入一个环保团体或俱乐部。	**1**	**2**	**3**	**4**	**5**	**6**	**7**
3. 我愿意缴纳更多的个人所得税来支持政府用于环境治理。	**1**	**2**	**3**	**4**	**5**	**6**	**7**
4. 如果能够让空气变得更加洁净，我愿意支付更多的电费。	**1**	**2**	**3**	**4**	**5**	**6**	**7**
5. 如果我经常购物的公司涉嫌环境污染，我将不再光顾，即使这让我的生活变得不那么方便。	**1**	**2**	**3**	**4**	**5**	**6**	**7**
6. 我愿意做出个人牺牲来减少环境污染，即使见效不那么快。	**1**	**2**	**3**	**4**	**5**	**6**	**7**

【亲人版】

1、请你集中精神想像特定的一个**亲人**____________（提供称谓：如爸爸、妈妈、叔叔、姑姑、姨妈、哥哥等）作为本次实验的目标人物，即**一个你最亲密的亲人，你们无话不谈，关系非常要好，是对你来说一个非常非常重要的人。**

2、下图为 7 对重叠程度不同的圆圈，一个代表**自己**，另一个代表你的**亲人**，交叉部分代表的是自我和他人重叠的程度（自我-他人重叠是指交往关系中的个体不同程度地接受和理解他人的观念、资源，从而出现自我和他人信息表征的重叠现象）。请在下面的图形中选择最符合**你和亲人**关系的图形（打“√”）。

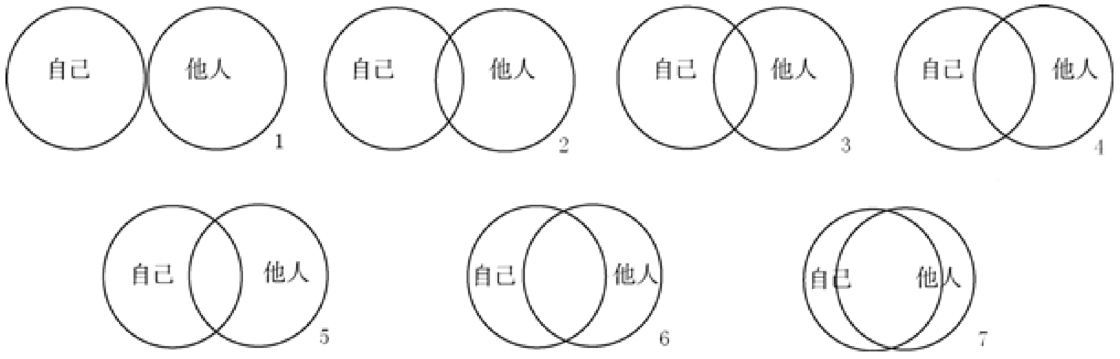

问卷 1

首先，请阅读下列在现实生活中可能遇到的一个情境，仔细阅读这个情境中发生的故事，尽可能想象你的**亲人**是该情境中的主人公，当这个情境在你脑海中比较清楚时，请你按要求完成后面的问题。

情境 1：________（亲人称谓）家里有很多垃圾需要丢弃，现在需要将其分离并带到垃圾收集点。_________（亲人称谓）不耐烦地把不同种类的垃圾分开，并把它们都扔在一个大袋子里。然后，_______（亲人称谓）走到垃圾收集站，看到剩余的垃圾桶已经满了，就把装着各种垃圾的大袋子扔进了厨余垃圾桶里。

请问：<u>他/她做了什么？</u>

____

____

____

情境 2：__________（亲人称谓）经营着一个化工厂，化工厂最近因赶进度而在短时间内产生了较多的污水排放，如果要控制污水排放量势必会增加额外的经费投入。尽管厂里有人提议及时进行净化处理，但_______（亲人称谓）作为工厂决策人，思量再三，还是决定放任污水排放，追求最大化经济利益。

请问：<u>他/她做了什么？</u>

____

____

____

（2）该行为属于环境不友好的行为吗？（1 = 是，2 = 否,打√）。

（3）请评估该行为的环保程度（1 = 极其不环保，2 = 有点不环保；3 = 不确定；4 = 非常环保；5 = 绝对环保；打√）。

问卷 2

请您根据上面两个情境，再思考与回忆一下你的亲人之前还有做过类似的**环境不友好行为**的经历吗？请花几分钟时间回忆与组织语言，并写下一次**亲人做过的、让你最难忘的环境不友好行为的事情（与你无关的）**，要求不少于 100 字。

请问：（1）<u>他/她的行为对你有什么影响？</u>

____

____

____

（2）该行为属于环境不友好的行为吗？（1 = 是，2 = 否,打√）。

（3）请评估该行为的环保程度（1 = 极其不环保，2 = 有点不环保；3 = 不确定；4 = 非常环保；5 = 绝对环保；打√）

问卷 3

请您根据自己的真实感受与意愿，对下列 6 个条目的内容进行主观意愿的等级评定（打“√”），其中 1 代表完全不同意，7 代表完全同意。该量表的结果评定无好坏之分，只需要您真实反应填写即可。

项 目	1=完全不同意 —————— 7=完全同意						
1. 我愿意签署一份请愿书来支持环保事业。	1	2	3	4	5	6	7
2. 我会考虑加入一个环保团体或俱乐部。	1	2	3	4	5	6	7
3. 我愿意缴纳更多的个人所得税来支持政府用于环境治理。	1	2	3	4	5	6	7
4. 如果能够让空气变得更加洁净，我愿意支付更多的电费。	1	2	3	4	5	6	7
5. 如果我经常购物的公司涉嫌环境污染，我将不再光顾，即使这让我的生活变得不那么方便。	1	2	3	4	5	6	7
6. 我愿意做出个人牺牲来减少环境污染，即使见效不那么快。	1	2	3	4	5	6	7

【恋人版】

1、请你集中精神想像您的**恋人**____________（提供昵称）作为本次实验的目标人物。

2、下图为 7 对重叠程度不同的圆圈，一个代表**自己**，另一个代表你的**恋人**，交叉部分代表的是自我和他人重叠的程度（自我-他人重叠是指交往关系中的个体不同程度地接受和理解他人的观念、资源，从而出现自我和他人信息表征的重叠现象）。请在下面的图形中选择最符合**你和恋人**关系的图形（打“√”）。

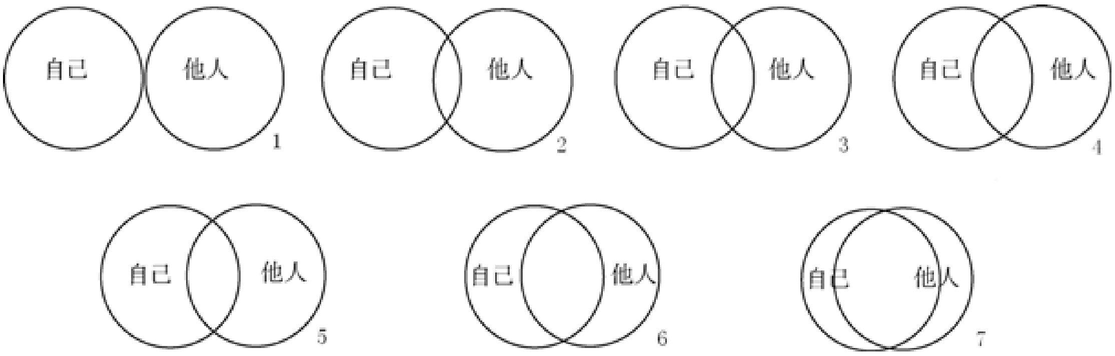

问卷 1

首先，请阅读下列在现实生活中可能遇到的一个情境，仔细阅读这个情境中发生的故事，尽可能想象你的**恋人**是该情境中的主人公，当这个情境在你脑海中比较清楚时，请你按要求完成后面的问题。

情境 1：________（恋人昵称）家里有很多垃圾需要丢弃，现在需要将其分离并带到垃圾收集点。_________（恋人昵称）不耐烦地把不同种类的垃圾分开，并把它们都扔在一个大袋子里。然后，_______（恋人昵称）走到垃圾收集站，看到剩余的垃圾桶已经满了，就把装着各种垃圾的大袋子扔进了厨余垃圾桶里。

请问：<u>他/她做了什么？</u>

____

____

____

情境 2：学校第五教学楼附近有一个湖泊，来来往往的学生很多，_________（恋人昵称）刚好在附近有美术课，每次上完课同学们都争抢着去洗手间清洗用具，因为嫌排队太麻烦，_______（恋人昵称）好几次将美术课未用完的颜料墨水倒进湖中，路过的同学指指点点，_________（恋人昵称）依然我行我素，不为所动。

请问：<u>他/她做了什么？</u>

____

____

____

问卷 2

请您根据上面两个情境，再思考与回忆一下你的**恋人**之前还有做过类似的**环境不友好行为**的经历吗？请花几分钟时间回忆与组织语言，并写下一次**恋人做过的、让你最难忘的环境不友好行为的事情（与你无关的）**，要求不少于 100 字。

____

____

____

____

____

____

____

____

____

____

____

____

____

____

____

请问：（1）他/她的行为对你有什么影响？

____

____

____

（2）该行为属于环境不友好的行为吗？（1 = 是，2 = 否,打√）。

（3）请评估该行为的环保程度（1 = 极其不环保，2 = 有点不环保；3 = 不确定；4 = 非常环保；5 = 绝对环保；打√）

问卷 3

请您根据自己的真实感受与意愿，对下列 6 个条目的内容进行主观意愿的等级评定（打“√”），其中 1 代表完全不同意，7 代表完全同意。该量表的结果评定无好坏之分，只需要您真实反应填写即可。

项 目	**1=完全不同意 ——————— 7=完全同意**						
1. 我愿意签署一份请愿书来支持环保事业。	**1**	**2**	**3**	**4**	**5**	**6**	**7**
2. 我会考虑加入一个环保团体或俱乐部。	**1**	**2**	**3**	**4**	**5**	**6**	**7**
3. 我愿意缴纳更多的个人所得税来支持政府用于环境治理。	**1**	**2**	**3**	**4**	**5**	**6**	**7**
4. 如果能够让空气变得更加洁净，我愿意支付更多的电费。	**1**	**2**	**3**	**4**	**5**	**6**	**7**
5. 如果我经常购物的公司涉嫌环境污染，我将不再光顾，即使这让我的生活变得不那么方便。	**1**	**2**	**3**	**4**	**5**	**6**	**7**
6. 我愿意做出个人牺牲来减少环境污染，即使见效不那么快。	**1**	**2**	**3**	**4**	**5**	**6**	**7**

问卷 4（RISC）

请您根据自己的真实感受与意愿，对下列 9 个条目的内容进行主观意愿的等级评定（打“√”），其中 1 代表完全不同意，7 代表完全同意。该量表的结果评定无好坏之分，只需要您真实反应填写即可。

1=完全不同意 2=不同意 3=有点不同意 4=不确定 5=有点同意 6=同意 7=完全同意

此处的亲近关系指与亲近他人的关系，即与父母、朋友、恋人等的关系。

题 目	1=完全不同意 ———————— 7=完全同意						
1. 我的亲近关系是“我是什么样的人”的重要反映。	1	2	3	4	5	6	7
2. 当我和某人很亲近时，我感觉他是“我”的重要组成部分。	1	2	3	4	5	6	7
3. 我常常为我朋友的重大成就而自豪。	1	2	3	4	5	6	7
4. 我认为通过观察和了解我的好朋友可以获得我的某些重要特征。	1	2	3	4	5	6	7
5. 当我审视自己时，我通常会同时想起我的密友或家人。	1	2	3	4	5	6	7
6. 如果有人伤害了我亲近的人，我会感觉自己也受到了伤害。	1	2	3	4	5	6	7
7. 一般来说，我的亲近关系是自我形象的重要组成部分。	1	2	3	4	5	6	7
8. 我的自豪感来自于我了解我的挚友。	1	2	3	4	5	6	7
9. 当我与某人建立亲近关系时，我通常会非常认同这个人。	1	2	3	4	5	6	7

问卷 5

请您根据自己的真实感受与意愿，对下列 3 个条目的内容进行主观意愿的等级评定，其中 1 代表完全不同意，7 代表完全同意。该量表的结果评定无好坏之分，只需要您真实反应填写即可。

1=完全不同意 2=不同意 3=有点不同意 4=不确定 5=有点同意 6=同意 7=完全同意

**题　目**	**1=完全不同意 ———————— 7=完全同意**						
1. 凡事注重环保是我个人的重要特质。	**1**	**2**	**3**	**4**	**5**	**6**	**7**
2. 我属于一举一动都很注重环保的人。	**1**	**2**	**3**	**4**	**5**	**6**	**7**
3. 我认为自己是一个环保的人。	**1**	**2**	**3**	**4**	**5**	**6**	**7**
