# Supplementary material for: Breaking the vicarious trap: two buffers against close others’ environmental influence
Source: BMC Psychol. 2026 Oct 2;14:1449. doi: 10.1186/s40359-026-05363-y (PMC13644283; doi:10.1186/s40359-026-05363-y)
Supplement: Supplementary file 1 [file 40359_2026_5363_MOESM1_ESM.zip › materials and data/materials/Study 1/1回忆想象任务-环保友好组.pdf]

亲爱的同学：

您好！非常感谢您抽取宝贵的时间来参与本次实验研究，您填写的每一个答案对我们来说都十分重要，希望您认真作答。本研究所得数据仅供科学研究之用并严格保密，请您放心作答，感谢您的支持与帮助！

个人情况

年龄：　　　　　　　　　　　　　　　性别：

恋爱时长：（　　）个月

【自我版】

问卷 1

首先，请阅读下列在现实生活中可能遇到的一个情境，仔细阅读这个情境中发生的故事，尽可能想象你**自己**是该情境中的主人公，当这个情境在你脑海中比较清楚时，请你按要求完成后面的问题。

情境 1：我家中有大量的垃圾，现在需要将其分离并带到垃圾收集点。尽管小区并没有强制要求，但我是一个环保主义的践行者，你认真地将塑料、纸张、厨余垃圾和玻璃瓶等进行分离，在此过程中因为干湿垃圾杂糅在一起，颇费了一番功夫。最终，我按照厨余垃圾、其他垃圾、可回收物、有害垃圾四种类型将相应垃圾丢弃到了指定的垃圾箱中。

请问：（1）你做了什么？

____

____

____

（2）该行为属于环境友好的行为吗？（1 = 是，2 = 否,打√）。

（3）请评估该行为的环保程度（1 = 极其不环保，2 = 有点不环保；3 = 不确定；4 = 非常环保；5 = 绝对环保；打√）。

情境 2：某公益环保机构来到你们学校组织了一场“绿水青山”环保主题的公益捐赠活动，作为环保行动的积极倡导者，我来到志愿者和捐款箱面前，想了想这个月仅剩不多的生活费，思虑片刻后，还是决定将钱都捐赠出去用于环保事业。

请问：（1）你做了什么？

____

____

____

（2）该行为属于环境友好的行为吗？（1 = 是，2 = 否,打√）。

（3）请评估该行为的环保程度（1 = 极其不环保，2 = 有点不环保；3 = 不确定；4 = 非常环保；5 = 绝对环保；打√）

问卷 2

请您根据上面两个情境，再思考与回忆一下你**自己**之前还有做过类似的**环境友好行为**的经历吗？请花几分钟时间回忆与组织语言，并用笔写下一次**你做过的、让你最难忘的环境友好行为的事情**，要求不少于 100 字。

____

____

____

____

____

____

____

____

____

____

请问：（1）<u>你过去的行为对你现在有什么影响？</u>

____

____

____

（2）该行为属于环境友好的行为吗？（1 = 是，2 = 否，打√）。

（3）请评估该行为的环保程度（1 = 极其不环保，2 = 有点不环保；3 = 不确定；4 = 非常环保；5 = 绝对环保；打√）

问卷 3

请您根据自己的真实感受与意愿，对下列 6 个条目的内容进行主观意愿的等级评定（打“√”），其中 1 代表完全不同意，7 代表完全同意。该量表的结果评定无好坏之分，只需要您真实反应填写即可。

项 目	1=完全不同意 —————— 7=完全同意						
1. 我愿意签署一份请愿书来支持环保事业。	**1**	**2**	**3**	**4**	**5**	**6**	**7**
2. 我会考虑加入一个环保团体或俱乐部。	**1**	**2**	**3**	**4**	**5**	**6**	**7**
3. 我愿意缴纳更多的个人所得税来支持政府用于环境治理。	**1**	**2**	**3**	**4**	**5**	**6**	**7**
4. 如果能够让空气变得更加洁净，我愿意支付更多的电费。	**1**	**2**	**3**	**4**	**5**	**6**	**7**
5. 如果我经常购物的公司涉嫌环境污染，我将不再光顾，即使这让我的生活变得不那么方便。	**1**	**2**	**3**	**4**	**5**	**6**	**7**
6. 我愿意做出个人牺牲来减少环境污染，即使见效不那么快。	**1**	**2**	**3**	**4**	**5**	**6**	**7**

【亲人版】

1、请你集中精神想像特定的一个**亲人**_____________（提供称谓：如爸爸、妈妈、叔叔、姑姑、姨妈、哥哥等）作为本次实验的目标人物，即**一个你最亲密的亲人，你们无话不谈，关系非常要好，是对你来说一个非常非常重要的人。**

2、下图为 7 对重叠程度不同的圆圈，一个代表**自己**，另一个代表你的**亲人**，交叉部分代表的是自我和他人重叠的程度（自我-他人重叠是指交往关系中自我与他人关系的亲密程度，以及两者在观念和态度上的相似程度，得分越高，代表亲密度越高）。请在下面的图形中选择最符合**你和亲人**关系的图形（打“√”）。

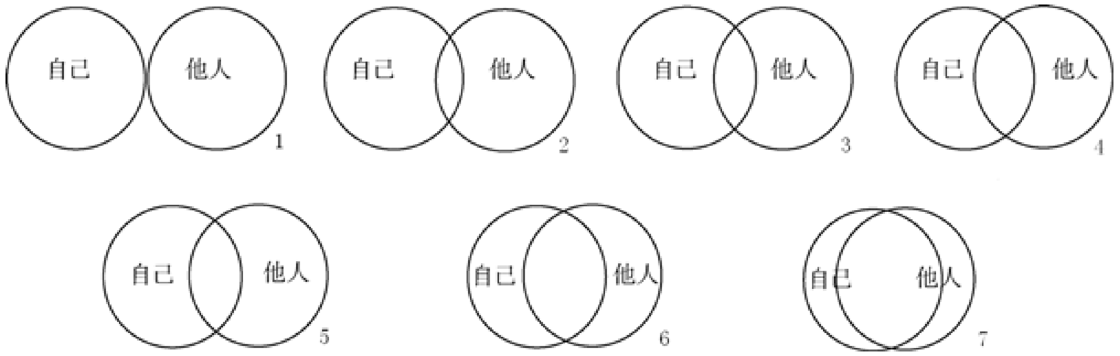

问卷 1

首先，请阅读下列在现实生活中可能遇到的一个情境，仔细阅读这个情境中发生的故事，尽可能想象你的**亲人**是该情境中的主人公，当这个情境在你脑海中比较清楚时，请你按要求完成后面的问题。

情境 1：________（亲人称谓）家中有大量的垃圾，现在需要将其分离并带到垃圾收集点。尽管小区并没有强制要求，但________（亲人称谓）是一个环保主义的践行者，他/她认真地将塑料、纸张、厨余垃圾和玻璃瓶等进行分离，在此过程中因为干湿垃圾杂糅在一起，颇费了一番功夫。最终，________（亲人称谓）按照厨余垃圾、其他垃圾、可回收物、有害垃圾四种类型将相应垃圾丢弃到了指定的垃圾箱中。

请问：他/她做了什么？

____

____

情境 2：________（亲人称谓）将要购买一个新冰箱，他/她的目光集中在两个容量相同，但价格和节能不同的冰箱上，其中一个价格便宜但不太节能，而另一个更节能但却价格更贵。__________（亲人称谓）权衡之后决定购买节能冰箱，虽然价格更贵，但它更有利于环境。

请问：（1）他/她做了什么？

____

____

（2）该行为属于环境友好的行为吗？（1 = 是，2 = 否,打√）。

（3）请评估该行为的环保程度（1 = 极其不环保，2 = 有点不环保；3 = 不确定；4 = 非常环保；5 = 绝对环保；打√）

问卷 2

请您根据上面两个情境，再思考与回忆一下你的亲人之前还有做过类似的**环境友好行为**的经历吗？请花几分钟时间回忆与组织语言，并用笔写下一次**亲人做过的、让你最难忘的环境友好行为的事情（与你无关的）**，要求不少于 100 字。

请问：（1）<u>他/她的行为对你有什么影响？</u>

（2）该行为属于环境友好的行为吗？（1 = 是，2 = 否,打√）。

（3）请评估该行为的环保程度（1 = 极其不环保，2 = 有点不环保；3 = 不确定；4 = 非常环保；5 = 绝对环保；打√）

问卷 3

请您根据自己的真实感受与意愿，对下列 6 个条目的内容进行主观意愿的等级评定（打“√”），其中 1 代表完全不同意，7 代表完全同意。该量表的结果评定无好坏之分，只需要您真实反应填写即可。

项 目	1=完全不同意 —————— 7=完全同意						
1. 我愿意签署一份请愿书来支持环保事业。	**1**	**2**	**3**	**4**	**5**	**6**	**7**
2. 我会考虑加入一个环保团体或俱乐部。	**1**	**2**	**3**	**4**	**5**	**6**	**7**
3. 我愿意缴纳更多的个人所得税来支持政府用于环境治理。	**1**	**2**	**3**	**4**	**5**	**6**	**7**
4. 如果能够让空气变得更加洁净，我愿意支付更多的电费。	**1**	**2**	**3**	**4**	**5**	**6**	**7**
5. 如果我经常购物的公司涉嫌环境污染，我将不再光顾，即使这让我的生活变得不那么方便。	**1**	**2**	**3**	**4**	**5**	**6**	**7**
6. 我愿意做出个人牺牲来减少环境污染，即使见效不那么快。	**1**	**2**	**3**	**4**	**5**	**6**	**7**

【恋人版】

1、请你集中精神想像您的**恋人**_____________（提供昵称）作为本次实验的目标人物。

2、下图为 7 对重叠程度不同的圆圈，一个代表**自己**，另一个代表你的**恋人**，交叉部分代表的是自我和他人重叠的程度（自我-他人重叠是指交往关系中自我与他人关系的亲密程度，以及两者在观念和态度上的相似程度，得分越高，代表亲密度越高）。请在下面的图形中选择最符合**你和恋人**关系的图形（打“√”）。

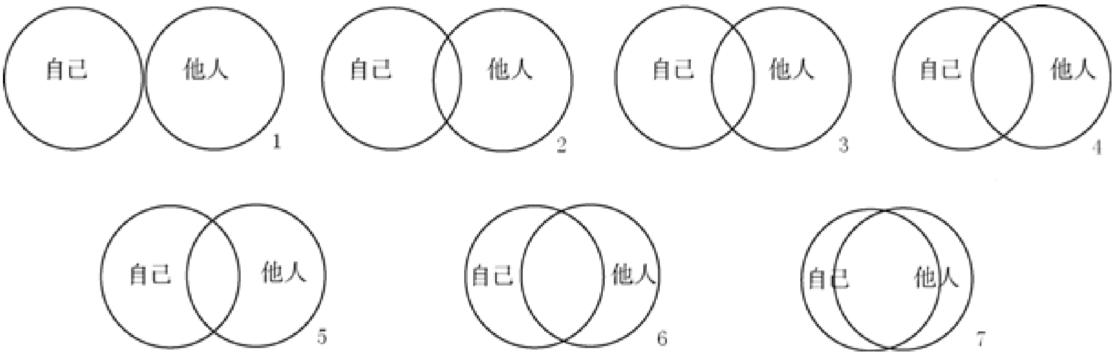

问卷 1

首先，请阅读下列在现实生活中可能遇到的一个情境，仔细阅读这个情境中发生的故事，尽可能想象你亲密的**恋人**是该情境中的主人公，当这个情境在你脑海中比较清楚时，请你按要求完成后面的问题。

情境 1：________（恋人昵称）家中有大量的垃圾，现在需要将其分离并带到垃圾收集点。尽管小区并没有强制要求，但________（恋人昵称）是一个环保主义的践行者，他/她认真地将塑料、纸张、厨余垃圾和玻璃瓶等进行分离，在此过程中因为干湿垃圾杂糅在一起，颇费了一番功夫。最终，________（恋人昵称）按照厨余垃圾、其他垃圾、可回收物、有害垃圾四种类型将相应垃圾丢弃到了指定的垃圾箱中。

请问：他/她做了什么？

____

____

____

情境 2：某公益环保机构来到你们学校组织了一场“绿水青山”环保主题的公益捐赠活动，作为环保行动的积极倡导者，________（恋人昵称）来到志愿者和捐款箱面前，想了想这个月仅剩不多的生活费，思虑片刻后，还是决定将钱都捐赠出去用于环保事业。

请问：他/她做了什么？

____

____

____

问卷 2

请您根据上面两个情境，再思考与回忆一下你的**恋人**之前还有做过类似的**环境友好行为**的经历吗？请花几分钟时间回忆与组织语言，并用笔写下一次**恋人做过的、让你最难忘的环境友好行为的事情（与你无关的）**，要求不少于 100 字。

请问：（1）他/她的行为对你有什么影响？

（2）该行为属于环境友好的行为吗？（1 = 是，2 = 否,打√）。

（3）请评估该行为的环保程度（1 = 极其不环保，2 = 有点不环保；3 = 不确定；4 = 非常环保；5 = 绝对环保；打√）

问卷 3

请您根据自己的真实感受与意愿，对下列 6 个条目的内容进行主观意愿的等级评定（打“√”），其中 1 代表完全不同意，7 代表完全同意。该量表的结果评定无好坏之分，只需要您真实反应填写即可。

项 目	1=完全不同意 ———————— 7=完全同意						
1. 我愿意签署一份请愿书来支持环保事业。	**1**	**2**	**3**	**4**	**5**	**6**	**7**
2. 我会考虑加入一个环保团体或俱乐部。	**1**	**2**	**3**	**4**	**5**	**6**	**7**
3. 我愿意缴纳更多的个人所得税来支持政府用于环境治理。	**1**	**2**	**3**	**4**	**5**	**6**	**7**
4. 如果能够让空气变得更加洁净，我愿意支付更多的电费。	**1**	**2**	**3**	**4**	**5**	**6**	**7**
5. 如果我经常购物的公司涉嫌环境污染，我将不再光顾，即使这让我的生活变得不那么方便。	**1**	**2**	**3**	**4**	**5**	**6**	**7**
6. 我愿意做出个人牺牲来减少环境污染，即使见效不那么快。	**1**	**2**	**3**	**4**	**5**	**6**	**7**

问卷 4（RISC）

请您根据自己的真实感受与意愿，对下列 9 个条目的内容进行主观意愿的等级评定（打“√”），其中 1 代表完全不同意，7 代表完全同意。该量表的结果评定无好坏之分，只需要您真实反应填写即可。

1=完全不同意 2=不同意 3=有点不同意 4=不确定 5=有点同意 6=同意 7=完全同意

此处的亲近关系指与亲近他人的关系，即与父母、朋友、恋人等的关系。

题　目	1=完全不同意 ———————— 7=完全同意						
1. 我的亲近关系是“我是什么样的人”的重要反映。	1	2	3	4	5	6	7
2. 当我和某人很亲近时，我感觉他是“我”的重要组成部分。	1	2	3	4	5	6	7
3. 我常常为我朋友的重大成就而自豪。	1	2	3	4	5	6	7
4. 我认为通过观察和了解我的好朋友可以获得我的某些重要特征。	1	2	3	4	5	6	7
5. 当我审视自己时，我通常会同时想起我的密友或家人。	1	2	3	4	5	6	7
6. 如果有人伤害了我亲近的人，我会感觉自己也受到了伤害。	1	2	3	4	5	6	7
7. 一般来说，我的亲近关系是自我形象的重要组成部分。	1	2	3	4	5	6	7
8. 我的自豪感来自于我了解我的挚友。	1	2	3	4	5	6	7
9. 当我与某人建立亲近关系时，我通常会非常认同这个人。	1	2	3	4	5	6	7

问卷 5

请您根据自己的真实感受与意愿，对下列 3 个条目的内容进行主观意愿的等级评定，其中 1 代表完全不同意，7 代表完全同意。该量表的结果评定无好坏之分，只需要您真实反应填写即可。

1=完全不同意 2=不同意 3=有点不同意 4=不确定 5=有点同意 6=同意 7=完全同意

**题 目**	**1=完全不同意 ——————— 7=完全同意**						
1. 凡事注重环保是我个人的重要特质。	**1**	**2**	**3**	**4**	**5**	**6**	**7**
2. 我属于一举一动都很注重环保的人。	**1**	**2**	**3**	**4**	**5**	**6**	**7**
3. 我认为自己是一个环保的人。	**1**	**2**	**3**	**4**	**5**	**6**	**7**
