# Supplementary material for: Breaking the vicarious trap: two buffers against close others’ environmental influence
Source: BMC Psychol. 2026 Oct 2;14:1449. doi: 10.1186/s40359-026-05363-y (PMC13644283; doi:10.1186/s40359-026-05363-y)
Supplement: Supplementary file 1 [file 40359_2026_5363_MOESM1_ESM.zip › materials and data/materials/Study 2/1回忆想象任务-环保友好组-公开(第一部分).pdf]

亲爱的同学：

您好！非常感谢您抽取宝贵的时间来参与本次实验研究，您填写的每一个答案对我们来说都十分重要，希望您认真作答。本研究所得数据仅供科学研究之用并严格保密，请您放心作答，感谢您的支持与帮助！

【第一部分】

问卷 1

请选择最符合你情况的选项，并在相应数字上打"√"。

1=完全不同意，7=完全同意

1. 凡事注重环保是我个人的重要特质。	1	2	3	4	5	6	7
2. 我属于一举一动都很注重环保的人。	1	2	3	4	5	6	7
3. 我认为自己是一个环保的人。	1	2	3	4	5	6	7

问卷 2

1、请你集中精神想像您的**密友**_____________（**提供姓名**）作为本次实验的目标人物，即**一个你最亲密的朋友，你们无话不谈，关系非常要好，是对你来说一个非常非常重要的人。**

2、下图为 7 对重叠程度不同的圆圈，一个代表**自己**，另一个代表你的**密友**，交叉部分代表的是自我和他人重叠的程度（自我-他人重叠是指交往关系中的个体不同程度地接受和理解他人的观念、资源，从而出现自我和他人信息表征的重叠现象）。请在下面的图形中选择最符合**你和密友**关系的图形（打"√"）。

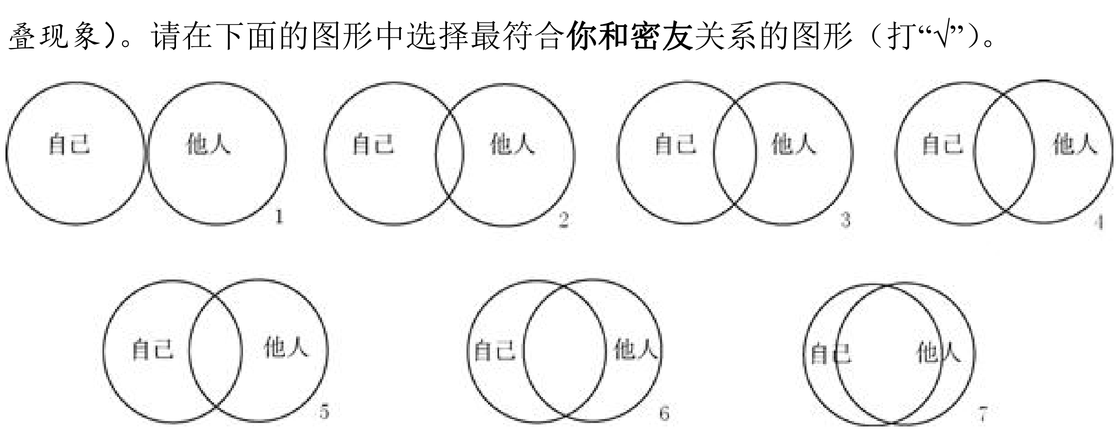

问卷 3

首先，请阅读下列在现实生活中可能遇到的一个情境，仔细阅读这个情境中发生的故事，尽可能想象你亲密的**密友**是该情境中的主人公，当这个情境在你脑海中比较清楚时，请你按要求完成后面的问题。

情境 1：________（密友姓名）**家中有大量的垃圾，现在需要将其分离并带到垃圾收集点。尽管小区并没有强制要求，但**________（密友姓名）**是一个环保主义的践行者，他/她认真地将塑料、纸张、厨余垃圾和玻璃瓶等进行分离，在此过程中因为干湿垃圾杂糅在一起，颇费了一番功夫。最终，**________（密友姓名）**按照厨余垃圾、其他垃圾、可回收物、有害垃圾四种类型将相应垃圾丢弃到了指定的垃圾箱中。**

请问：他/她做了什么？

____

____

____

情境 2：某公益环保机构来到你们学校组织了一场"绿水青山"环保主题的公益捐赠活动，作为环保行动的积极倡导者，________（密友姓名）**来到志愿者和捐款箱面前，想了想这个月仅剩不多的生活费，思虑片刻后，还是决定将钱都捐赠出去用于环保事业。**

请问：他/她做了什么？

____

____

____

问卷 4

请您根据上面两个情境，再思考与回忆一下你的**密友**之前还有做过类似的**环保行为**的经历吗？请花几分钟时间回忆与组织语言，并用笔写下**密友做过的、让你最难忘的环保行为（与你无关的）**，要求不少于 100 字。

____

____

____

____

____

____

____

____

____

____

____

____

请问：（1）<u>他/她的行为对你有什么影响？</u>

____

____

____

（2）该行为属于环保行为吗？（1＝ 是，2＝ 否，打√）。

（3）请评估该行为的环保程度（1＝ 极其不环保，2＝ 有点不环保，3＝ 不确定，4＝ 非常环保，5＝ 绝对环保，打√）

【个人信息】

姓名：	性别：
年龄：	联系方式：
