# Supplementary material for: Breaking the vicarious trap: two buffers against close others’ environmental influence
Source: BMC Psychol. 2026 Oct 2;14:1449. doi: 10.1186/s40359-026-05363-y (PMC13644283; doi:10.1186/s40359-026-05363-y)
Supplement: Supplementary file 1 [file 40359_2026_5363_MOESM1_ESM.zip › materials and data/materials/Study 2/2回忆想象任务-环保友好组-公开(第二部分).pdf]

【第二部分】

问卷 5

（1）在这件事发生后，它在多大程度上改变了你对你的密友道德或不道德程度的认知，请在符合的数字上打“√”。

0 代表没有改变，越往左边表示越不道德，越往右边表示越道德。														
-7	-6	-5	-4	-3	-2	-1	0	1	2	3	4	5	6	7

（2）在这件事发生后，它在多大程度上改变了你对你自己道德或不道德程度的认知，请在符合的数字上打“√”。

0 代表没有改变，越往左边表示越不道德，越往右边表示越道德。														
-7	-6	-5	-4	-3	-2	-1	0	1	2	3	4	5	6	7

问卷 6

序号	在这件事发生后，你感觉到多少以下情绪（打“√”）？1（完全没有）——7（非常多）							
1	愤 怒	1	2	3	4	5	6	7
2	厌 恶	1	2	3	4	5	6	7
3	蔑 视	1	2	3	4	5	6	7
4	羞 耻	1	2	3	4	5	6	7
5	内 疚	1	2	3	4	5	6	7
6	尴 尬	1	2	3	4	5	6	7
7	自 豪	1	2	3	4	5	6	7
8	钦 佩	1	2	3	4	5	6	7
9	快 乐	1	2	3	4	5	6	7
10	沮 丧	1	2	3	4	5	6	7
11	同 情	1	2	3	4	5	6	7

问卷 7

请选择最符合你情况的选项，并在相应数字上打“√”。

1=完全不同意，7=完全同意

1. 我不在乎别人怎么看我。	**1**	**2**	**3**	**4**	**5**	**6**	**7**
2. 我想以积极正面的方式向他人呈现自己。	**1**	**2**	**3**	**4**	**5**	**6**	**7**
3. 我想给他人留下积极正面的形象。	**1**	**2**	**3**	**4**	**5**	**6**	**7**
4. 我想使自己对他人来说看上去不错。	**1**	**2**	**3**	**4**	**5**	**6**	**7**

问卷 8

请选择最符合你情况的选项，并在相应数字上打“√”。

1=完全不同意，7=完全同意

1. 当我从事环保行为时，我觉得我是一个有能力的人。	**1**	**2**	**3**	**4**	**5**	**6**	**7**
2. 当我从事环保行为时，我经常觉得自己能力不足。	**1**	**2**	**3**	**4**	**5**	**6**	**7**
3. 当我从事环保行为时，我觉得自己很能干，很有效率。	**1**	**2**	**3**	**4**	**5**	**6**	**7**
4. 当我从事环保行为时，我感到被爱和被关心。	**1**	**2**	**3**	**4**	**5**	**6**	**7**
5. 当我从事环保行为时，我常常感觉与他人有距离感。	**1**	**2**	**3**	**4**	**5**	**6**	**7**
6. 当我从事环保行为时，我感到与他人关系非常亲密。	**1**	**2**	**3**	**4**	**5**	**6**	**7**
7. 当我从事环保行为时，我可以自由地做我自己。	**1**	**2**	**3**	**4**	**5**	**6**	**7**
8. 当我从事环保行为时，我有发言权，可以表达我的意见。	**1**	**2**	**3**	**4**	**5**	**6**	**7**
9. 当我从事环保行为时，我在某些方面感到被控制和压力感。	**1**	**2**	**3**	**4**	**5**	**6**	**7**

问卷 9

请阅读下面这段材料，然后在相应数字上打“√”。

1=完全不同意，7=完全同意

随着春节临近，过年的气氛越来越浓，很多人开始讨论该过个“绿色春节”，少放鞭炮，以免污染空气。“达尔问自然求知社”发起不放或少放烟花爆竹的绿色过年活动，号召大家把省下的鞭炮钱捐出来，每人捐助 25 元或者 25 元的倍数金额，给所在城市的环保组织购买 PM2.5 监测仪，为春节不出现灰霾天气出一份力。

1. 绿色春节是减少资源消耗的好方法。	**1**	**2**	**3**	**4**	**5**	**6**	**7**
2. 这个活动将提高对资源消耗问题的认识。	**1**	**2**	**3**	**4**	**5**	**6**	**7**
3. 我认为这个活动是无用的。	**1**	**2**	**3**	**4**	**5**	**6**	**7**
4. 我认为这个活动很重要。	**1**	**2**	**3**	**4**	**5**	**6**	**7**
