# Supplementary material for: Breaking the vicarious trap: two buffers against close others’ environmental influence
Source: BMC Psychol. 2026 Oct 2;14:1449. doi: 10.1186/s40359-026-05363-y (PMC13644283; doi:10.1186/s40359-026-05363-y)
Supplement: Supplementary file 1 [file 40359_2026_5363_MOESM1_ESM.zip › materials and data/materials/Study 2/8回忆想象任务-环保不友好组-匿名.pdf]

亲爱的同学：

您好！非常感谢您抽取宝贵的时间来参与本次实验研究，您填写的每一个答案对我们来说都十分重要，希望您认真作答。本研究所得数据仅供科学研究之用并严格保密，请您放心作答，感谢您的支持与帮助！

【个人信息】

年龄：　　　　　　　　　性别：

【第一部分】

问卷 1

请选择最符合你情况的选项，并在相应数字上打“√”。

1=完全不同意，7=完全同意

1. 凡事注重环保是我个人的重要特质。	**1**	**2**	**3**	**4**	**5**	**6**	**7**
2. 我属于一举一动都很注重环保的人。	**1**	**2**	**3**	**4**	**5**	**6**	**7**
3. 我认为自己是一个环保的人。	**1**	**2**	**3**	**4**	**5**	**6**	**7**

问卷 2

1、请你集中精神想像您的**密友**_____________（**提供昵称**）作为本次实验的目标人物，即**一个你最亲密的朋友，你们无话不谈，关系非常要好，是对你来说一个非常非常重要的人。**

2、下图为 7 对重叠程度不同的圆圈，一个代表**自己**，另一个代表你的**密友**，交叉部分代表的是自我和他人重叠的程度（自我-他人重叠是指交往关系中的个体不同程度地接受和理解他人的观念、资源，从而出现自我和他人信息表征的重叠现象）。请在下面的图形中选择最符合**你和密友**关系的图形（打“√”）。

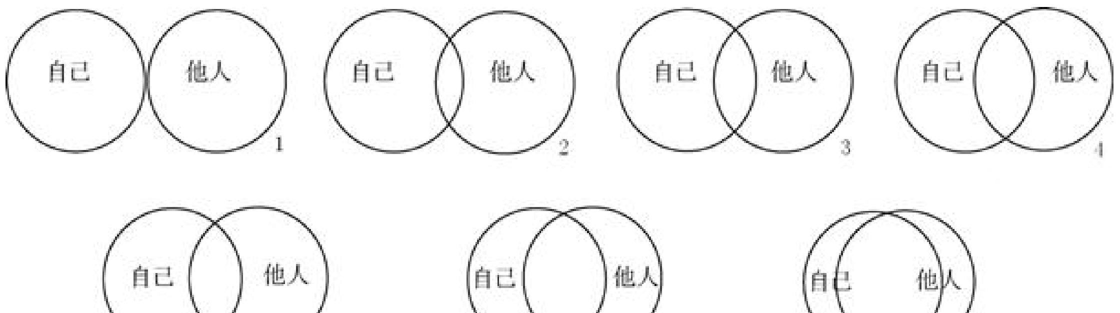

问卷 3

首先，请阅读下列在现实生活中可能遇到的一个情境，仔细阅读这个情境中发生的故事，尽可能想象你的**密友**是该情境中的主人公，当这个情境在你脑海中比较清楚时，请你按要求完成后面的问题。

情境 1：________（密友昵称）**家里有很多垃圾需要丢弃，现在需要将其分离并带到垃圾收集点。**________（密友昵称）**不耐烦地把不同种类的垃圾分开，并把它们都扔在一个大袋子里。然后，**_______（密友昵称）**走到垃圾收集站，看到剩余的垃圾桶已经满了，就把装着各种垃圾的大袋子扔进了厨余垃圾桶里。**

请问：他/她做了什么？

____

____

____

情境 2：学校第五教学楼附近有一个湖泊，来来往往的学生很多，_________（密友昵称）**刚好在附近有美术课，每次上完课同学们都争抢着去洗手间清洗用具，因为嫌排队太麻烦，**_______（密友昵称）**好几次将美术课未用完的颜料墨水倒进湖中，路过的同学指指点点，**_________（密友昵称）**依然我行我素，不为所动。**

请问：他/她做了什么？

____

____

____

问卷 4

请您根据上面两个情境，再思考与回忆一下你的**密友**之前还有做过类似的**不环保行为**的经历吗？请花几分钟时间回忆与组织语言，并写下**密友做过的、让你最难忘的不环保行为（与你无关的）**，要求不少于 100 字。

请问：（1）<u>他/她的行为对你有什么影响？</u>

（2）该行为属于环保行为吗？（1＝ 是，2＝ 否，打“√”）

（3）请评估该行为的环保程度（1＝ 极其不环保，2＝ 有点不环保，3＝ 不确定，4＝ 非常环保，5＝ 绝对环保，打“√”）

【第二部分】

问卷 5

（1）在这件事发生后，它在多大程度上改变了你对你的密友道德或不道德程度的认知，请在符合的数字上打“√”。

0 代表没有改变，越往左边表示越不道德，越往右边表示越道德。														
-7	-6	-5	-4	-3	-2	-1	0	1	2	3	4	5	6	7

（2）在这件事发生后，它在多大程度上改变了你对你自己道德或不道德程度的认知，请在符合的数字上打“√”。

0 代表没有改变，越往左边表示越不道德，越往右边表示越道德。														
-7	-6	-5	-4	-3	-2	-1	0	1	2	3	4	5	6	7

问卷 6

序号	在这件事发生后，你感觉到多少以下情绪（打“√”）？1（完全没有）——7（非常多）							
1	愤 怒	1	2	3	4	5	6	7
2	厌 恶	1	2	3	4	5	6	7
3	蔑 视	1	2	3	4	5	6	7
4	羞 耻	1	2	3	4	5	6	7
5	内 疚	1	2	3	4	5	6	7
6	尴 尬	1	2	3	4	5	6	7
7	自 豪	1	2	3	4	5	6	7
8	钦 佩	1	2	3	4	5	6	7
9	快 乐	1	2	3	4	5	6	7
10	沮 丧	1	2	3	4	5	6	7
11	同 情	1	2	3	4	5	6	7

问卷 7

请选择最符合你情况的选项，并在相应数字上打“√”。

1=完全不同意，7=完全同意

1. 我不在乎别人怎么看我。	**1**	**2**	**3**	**4**	**5**	**6**	**7**
2. 我想以积极正面的方式向他人呈现自己。	**1**	**2**	**3**	**4**	**5**	**6**	**7**
3. 我想给他人留下积极正面的形象。	**1**	**2**	**3**	**4**	**5**	**6**	**7**
4. 我想使自己对他人来说看上去不错。	**1**	**2**	**3**	**4**	**5**	**6**	**7**

问卷 8

请选择最符合你情况的选项，并在相应数字上打“√”。

1=完全不同意，7=完全同意

1. 当我从事环保行为时，我觉得我是一个有能力的人。	**1**	**2**	**3**	**4**	**5**	**6**	**7**
2. 当我从事环保行为时，我经常觉得自己能力不足。	**1**	**2**	**3**	**4**	**5**	**6**	**7**
3. 当我从事环保行为时，我觉得自己很能干，很有效率。	**1**	**2**	**3**	**4**	**5**	**6**	**7**
4. 当我从事环保行为时，我感到被爱和被关心。	**1**	**2**	**3**	**4**	**5**	**6**	**7**
5. 当我从事环保行为时，我常常感觉与他人有距离感。	**1**	**2**	**3**	**4**	**5**	**6**	**7**
6. 当我从事环保行为时，我感到与他人关系非常亲密。	**1**	**2**	**3**	**4**	**5**	**6**	**7**
7. 当我从事环保行为时，我可以自由地做我自己。	**1**	**2**	**3**	**4**	**5**	**6**	**7**
8. 当我从事环保行为时，我有发言权，可以表达我的意见。	**1**	**2**	**3**	**4**	**5**	**6**	**7**
9. 当我从事环保行为时，我在某些方面感到被控制和压力感。	**1**	**2**	**3**	**4**	**5**	**6**	**7**

问卷 9

请阅读下面这段材料，然后在相应数字上打“√”。

1=完全不同意，7=完全同意

随着春节临近，过年的气氛越来越浓，很多人开始讨论该过个“绿色春节”，少放鞭炮，以免污染空气。“达尔问自然求知社”发起不放或少放烟花爆竹的绿色过年活动，号召大家把省下的鞭炮钱捐出来，每人捐助 25 元或者 25 元的倍数金额，给所在城市的环保组织购买 PM2.5 监测仪，为春节不出现灰霾天气出一份力。

1. 绿色春节是减少资源消耗的好方法。	**1**	**2**	**3**	**4**	**5**	**6**	**7**
2. 这个活动将提高对资源消耗问题的认识。	**1**	**2**	**3**	**4**	**5**	**6**	**7**
3. 我认为这个活动是无用的。	**1**	**2**	**3**	**4**	**5**	**6**	**7**
4. 我认为这个活动很重要。	**1**	**2**	**3**	**4**	**5**	**6**	**7**
