# Supplementary material for: Breaking the vicarious trap: two buffers against close others’ environmental influence
Source: BMC Psychol. 2026 Oct 2;14:1449. doi: 10.1186/s40359-026-05363-y (PMC13644283; doi:10.1186/s40359-026-05363-y)
Supplement: Supplementary file 1 [file 40359_2026_5363_MOESM1_ESM.zip › materials and data/materials/Study 3/环境自我认同量表.docx]

**环境自我认同量表**

请选择最符合你情况的选项，并在相应数字上打“√”。

1=完全不同意，7=完全同意。

| 1. 凡事注重环保是我个人的重要特质。 | **1** | **2** | **3** | **4** | **5** | **6** | **7** |
| --- | --- | --- | --- | --- | --- | --- | --- |
| 2. 我属于一举一动都很注重环保的人。 | **1** | **2** | **3** | **4** | **5** | **6** | **7** |
| 3. 我认为自己是一个环保的人。 | **1** | **2** | **3** | **4** | **5** | **6** | **7** |
