# Supplementary material for: Breaking the vicarious trap: two buffers against close others’ environmental influence
Source: BMC Psychol. 2026 Oct 2;14:1449. doi: 10.1186/s40359-026-05363-y (PMC13644283; doi:10.1186/s40359-026-05363-y)
Supplement: Supplementary file 1 [file 40359_2026_5363_MOESM1_ESM.zip › materials and data/materials/Study 4/大学生环境行为日记法问卷.docx]

大学生环境行为日记法问卷

非常感谢您能来参加我们课题组关于【**大学生环境行为**】的科学研究。在未来连续的**30天**里，我们将每天**晚上9点**给您发送调查问卷的链接，邀请您在**晚上9点—11点**期间，对今天自己有关环境行为的感受和情况进行真实记录和评价。请您根据自己每天的真实感受，认真完成问卷中的每个条目，每次需要**20分钟**。当您连续认真坚持30天后，我们将向您支付**60元**的实验报酬。

**问卷（一）**

1.首先请你集中精神想像特定的一个**密友**_____________（昵称即可），即一个你最亲密的朋友，你们无话不谈，关系非常要好，是对你来说一个非常非常重要的人。**请您仔细观察和回忆您的密友今天做了_______件（至少1件）您认为属于环保的行为，事无巨细均可记录**。同时，请您从中挑选出一件让您感受最深、觉得最有意义的环保行为，并详细记录其发生的经过和您的切身感受，不少于100字。请仔细观察与记录，确保每天都能有所记录。谢谢！

**请您详细记录和描述密友今天从事的一件环保行为：**

_____________________________________________________________________

_____________________________________________________________________

_____________________________________________________________________

_____________________________________________________________________

_____________________________________________________________________

_____________________________________________________________________

**问卷（二）**

2.**请仔细回忆您自己今天做了_______件****（至少1件）您认为属于环保的行为，事无巨细均可记录。**同时，请您从中挑选出一件让您自己感受最深、觉得最有意义的环保行为，并详细记录其发生的经过和您的切身感受，不少于100字。

**请您详细记录和描述您自己今天从事的一件环保行为：**

_____________________________________________________________________

_____________________________________________________________________

_____________________________________________________________________

_____________________________________________________________________

_____________________________________________________________________

_____________________________________________________________________

_____________________________________________________________________

_____________________________________________________________________

_____________________________________________________________________

_____________________________________________________________________

_____________________________________________________________________

_____________________________________________________________________

_____________________________________________________________________

_____________________________________________________________________

_____________________________________________________________________

_____________________________________________________________________

_____________________________________________________________________

_____________________________________________________________________

_____________________________________________________________________

_____________________________________________________________________

**问卷（三）**

**请选择最符合你情况的选项，并在相应数字上打“√”。1=完全不同意，7=完全同意。**

| **环境自我决定需要问卷（4个）** | **1** | **2** | **3** | **4** | **5** | **6** | **7** |
| --- | --- | --- | --- | --- | --- | --- | --- |
| 1. 今天我觉得自己是一个有能力去从事环保的人。 |  |  |  |  |  |  |  |
| 2. 今天我从事环保行为后感觉很开心。 |  |  |  |  |  |  |  |
| 3. 今天我从事环保行为时感到被爱和关心。 |  |  |  |  |  |  |  |
| 4. 今天我从事环保行为时感到与他人关系更亲密了。 |  |  |  |  |  |  |  |
| **亲环境意愿量表（4个）** | **1** | **2** | **3** | **4** | **5** | **6** | **7** |
| 5. 今天我愿意签署一份承诺书来支持环保事业。 |  |  |  |  |  |  |  |
| 6. 今天我有考虑过加入一个环保团体或俱乐部。 |  |  |  |  |  |  |  |
| 7. 今天我愿意付出更多努力让周围环境变得更好。 |  |  |  |  |  |  |  |
| 8. 今天我愿意做出个人牺牲来减少周围的环境污染。 |  |  |  |  |  |  |  |
| **环境自我认同问卷（2个）** | **1** | **2** | **3** | **4** | **5** | **6** | **7** |
| 9. 今天我认为环保是一个比较重要的积极品质。 |  |  |  |  |  |  |  |
| 10. 今天我认为自己变得更加环保了。 |  |  |  |  |  |  |  |

**今天的问卷调查到此结束！感谢您的积极支持！明天再见！**
