# Supplementary material for: Breaking the vicarious trap: two buffers against close others’ environmental influence
Source: BMC Psychol. 2026 Oct 2;14:1449. doi: 10.1186/s40359-026-05363-y (PMC13644283; doi:10.1186/s40359-026-05363-y)
Supplement: Supplementary file 1 [file 40359_2026_5363_MOESM1_ESM.zip › materials and data/materials/Study 4/干预方案.doc]

“环保，还宝，让世界更好”团体干预方案

本方案从“认知普及、情感激发、行为实施”出发，期望通过环保游戏体验、环保教育宣讲、环保纪录片观看、环保实践活动等来提升大学生的环境自我认同水平，提高环保意识，为大学生践行亲环境行为提供有效干预。

**访谈提纲**

1、你喜欢参加这个团体辅导吗？为什么?

2、团体辅导中你印象最深的有哪些事或是哪些活动？为什么?

3、参加完此次团体辅导后，你觉得有哪些方面的收获呢?

4、参加完此次团体辅导后，你怎样看待环保，以后会怎样做?

**团体辅导反馈表**

1、在本次活动中我学习到了：
2、我的收获是：

**团体承诺书**

我承诺:

1、自愿参加本次团体活动。保证不缺席，不迟到，不中途离开。
2、保证在活动期间把注意力集中在团体中，不做与团体活动无关的事情。
3、愿意在团体中坦诚地谈论自己的一切，真实开放自己，愿意不断成长。
4、在团体活动中，我会与其他成员保持团结友爱的关系，不攻击、贬损任何成员，坦诚对待团体中每一位成员，相互尊重，学会倾听，经验分享，相互信赖。
5、对团体活动保守秘密，严格遵守保密原则，尊重其他成员个人经历和隐私权。
6、积极服从、配合领导者和同伴的安排，认真及时完成领导者布置的各项作业。

本人已经仔细阅读并充分了解本契约的内容。

成员签名:

领导者签名:

**环保承诺书**

节能减排，建设生态文明，这是对子孙后代和世界负责的庄重承诺。作为大学生应积极参与节能减排行动，为此，我庄重承诺：

①大力宣传党和政府的节能环保政策；

②不断提高资源意识和环境忧患意识，自觉树立人与自然和谐相处的理念；

③坚决同破坏生态文明的行为作斗争；

④以实际行动履行节能减排，如在学校里节约用水，使用绿色产品等。

承诺人：

时间：

**团辅方案大纲**

| 单元主题 | 团体辅导目标 | 活动内容 |
| --- | --- | --- |
| 我们在一起 | 1. 介绍本次团体辅导的目标、性质，签署团体承诺书； 2. 形成团队，促进成员间相互了解，增进熟悉度； 3. 形成小组团体规范，绘制海报； 4. 冥想放松，结束本次活动。 | 1. 滚雪球 2. 我的自画像 3. 我的期待 4. 冥想放松 |
| 你好，环保 | 1. 激发团体热情，引入环保概念； 2. 增加环保认知储备； 3. 通过趣味问答，进一步巩固环保知识。 | 1. 棒打薄情郎 2. 环保知多少 3. 环保知识竞赛 4. 冥想放松 |
| “碳”为观止 | 1. 活跃气氛，认识到大自然间千丝万缕的联系； 2. 触发情感共鸣。 | 1. 心有千千结 2. 纪录片观赏 3. 冥想放松 |
| 环保，还宝 | 1. 以趣味游戏引出垃圾分类主题； 2. 进一步巩固认知变化； 3. 沉浸式演绎环保情景剧，激发环保热情。 | 1. 趣味投篮 2. 垃圾分类知多少 3. 演员的诞生 4. 冥想放松 |
| 我是“碳”路人 | 1. 活跃气氛，体验破坏环境的消极后果； 2. 启发日常环保行为，提升环境自我认同； 3. 总结“环保卫士”实践活动，分享感悟，引导大学生积极践行环保。 | 1. 自食其果 2. 环保清单 3. 环保卫士 4. 冥想放松 |
| 依依惜离别 | 1. 增强成员间的沟通，增进彼此感情； 2. 强化团体活动效果，产生迁移。 | 1. 正念冥想 2. 忆往昔 3. 送祝福 4. 惜离别 |

**团辅干预详细方案**

第一单元：我们在一起

日期：2023年10月16日	时间：18:30—19:30/19:40—20:40

地点：教育学院团体辅导室	参与人员：17（14）名成员和1名带领者

| 活动主题 | 团队建立：我们在一起 | | |
| --- | --- | --- | --- |
| 活动目标 | 1. 介绍本次团体辅导的目标、性质，签署团体承诺书； 2. 通过“滚雪球”“我的自画像”形成团队，促进成员间相互了解，增进熟悉度； 3. 通过“我的期待”形成小组团体规范，绘制海报； 4. 冥想放松，结束本次活动。 | | |
| 活动准备 | 冥想音乐、白纸、签字笔、海报、彩笔、团体承诺书 | | |
| 活动名称 | 活动目标 | 活动内容 | 时间 |
| 滚雪球 | 加快成员间相互认识 | （1）第一个成员进行自我介绍包括姓名、专业、爱好等； （2）后一位成员以同样的方式进行自我介绍，并且要重复前面所有成员的个人信息。 | 15min |
| 我的自画像 | 促进成员间的进一步熟悉了解 | （1）写出自己的3个突出特点； （2）领导者统一回收； （3）领导者随机发放，成员们猜测拿到的是谁的信息卡。 | 15min |
| 我的期待 | 介绍环境自我认同，澄清目标 | （1）每个成员写出对团体的期待，小组分享交流； （2）总结并绘制团体规范海报； （3）签名。 | 20min |
| 冥想放松 | 放松身心，结束活动 | （1）分享感受； （2）全体成员跟随音乐舒缓身心。   作业：学习环保知识题库内容；“环保卫士”之每日打卡一件环保小事，以小组为单位，小组长负责打卡并收集成员相应环保行为（视频、照片或文字）。 | 10min |

第二单元：你好，环保

日期：2023年10月23日	时间：18:30—19:30/19:40—20:40

地点：教育学院团体辅导室	参与人员：17（14）名成员和1名带领者

| 活动主题 | 认知普及：你好，环保 | | |
| --- | --- | --- | --- |
| 活动目标 | 1. “棒打薄情郎”激发团体热情，引入环保概念； 2. 通过环保知识讲授，增加环保认知储备； 3. 通过“环保知识问答赛”，进一步巩固环保知识。 | | |
| 活动准备 | 冥想音乐、报纸卷、小贴纸、PPT课件、白纸、签字笔、小礼品 | | |
| 活动名称 | 活动目标 | 活动内容 | 时间 |
| 棒打薄情郎 | 引入环保元素，拉近成员间距离 | （1）填画胸卡：挑选一个自己最喜欢的环保昵称，填写在胸卡上，分组交流：为什么喜欢这个昵称？ （2）棒打薄情郎：分组围圈就座，选一人手执报纸卷成的“棒子”，喊出一位成员的昵称，被叫者左右两侧的成员要马上站起来，否则由被叫者给与当头一棒，“棒打薄情郎”，反复做，直到大家熟悉彼此的名字。 | 15min |
| 环保知多少 | 增加环保知识储备 | （1）介绍世界环境日与低碳生活； （2）了解当前的环境问题； （3）引导如何保护环境。 | 20min |
| 环保知识竞赛 | 巩固激发环保意识 | （1）分为必答题和抢答题； （2）每组必答题10题，组员轮流作答，满分10分，答错1题扣1分； （3）抢答题10题，答对1题加2分，答错扣2分。 （4）总分最高者即为获胜，获胜组成员获得环保小礼品。 | 15min |
| 冥想放松 | 放松身心，结束活动 | （3）分享感受； （4）全体成员跟随音乐舒缓身心。   作业：“环保卫士”之每日打卡一件环保小事。 | 10min |

第三单元：“碳”为观止

日期：2023年10月30日	时间：18:30—19:30/19:40—20:40

地点：教育学院团体辅导室	参与人员：17（14）名成员和1名带领者

| 活动主题 | 情感激发：“碳”为观止 | | |
| --- | --- | --- | --- |
| 活动目标 | 1. “心有千千结”活跃气氛，认识到大自然间环环相扣的联系； 2. 通过环保纪录片观赏，触发情感共鸣。 | | |
| 活动准备 | 冥想音乐、视频资源 | | |
| 活动名称 | 活动目标 | 活动内容 | 时间 |
| 心有千千结 | 活跃气氛，认识到大自然间千丝万缕的联系 | （1）所有成员围成一个圈，每个人双手交叉，去握同伴的手，但不许握自我相临的人的手，也不许两只手去握同一个人的双手； （2）所有人的手都交叉在一起后，在不松开手的情况下小组成员一齐想办法将这个交叉解开，到最终会发现我们解开后组成的是一个圆圈。 | 10min |
| 纪录片观赏 | 触发情感共鸣 | 观看环保纪录片《碳路森林》 | 50min |
| 冥想放松 | 放松身心，结束活动 | （1）分享感受； （2）全体成员跟随音乐舒缓身心。   作业：分组排练环保主题情景剧；“环保卫士”之每日打卡一件环保小事。 | 10min |

第四单元：环保，还宝

日期：2023年11月6日	时间：18:30—19:30/19:40—20:40

地点：教育学院团体辅导室	参与人员：17（14）名成员和1名带领者

| 活动主题 | 知情合一：环保，还宝 | | |
| --- | --- | --- | --- |
| 活动目标 | 1. “垃圾分类趣味投篮”以趣味游戏引出垃圾分类主题； 2. 通过垃圾分类知识讲授，进一步巩固认知变化； 3. 通过“演员的诞生”，沉浸式演绎情景剧，激发环保热情。 | | |
| 活动准备 | 冥想音乐、PPT课件、白纸坨、垃圾桶、环保小礼品 | | |
| 活动名称 | 活动目标 | 活动内容 | 时间 |
| 趣味投篮 | 活跃气氛，引入垃圾分类主题 | （1）三组同时PK； （2）在规定时间内将10个写有不同物品名称的纸球投入相应垃圾桶； （3）最先全部“投篮”正确即为获胜； （4）限时5分钟。 | 10min |
| 垃圾分类知多少 | 提升巩固环保认知资源 | （1）垃圾分类背景 （2）垃圾分类方法 （3）生活场景应用示例 | 20min |
| 演员的诞生 | 触发情感共鸣，激发环保热情 | 分组上台演绎情景剧 | 20min |
| 冥想放松 | 放松身心，结束活动 | （1）分享感受； （2）全体成员跟随音乐舒缓身心。   作业：“环保卫士”之每日打卡一件环保小事。 | 10min |

第五单元：我是“碳”路人

日期：2023年11月13日	时间：18:30—19:30/19:40—20:40

地点：教育学院团体辅导室	参与人员：17（14）名成员和1名带领者

| 活动主题 | 行为实施：我是“碳”路人 | | |
| --- | --- | --- | --- |
| 活动目标 | 1. “自食其果”活跃气氛，体验破坏环境的消极后果； 2. 通过列举“环保清单”，启发日常环保行为，提升环境自我认同感； 3. 通过总结“环保卫士”实践活动，分享感悟，引导大学生积极践行环保。 | | |
| 活动准备 | 冥想音乐、欢快音乐、香蕉、西红柿、盐、醋、胡椒粉、糖、黑色塑料袋、白纸、签字笔 | | |
| 活动名称 | 活动目标 | 活动内容 | 时间 |
| 自食其果 | 活跃气氛，体验资源破坏的消极后果 | （1）领导者把水果和佐料一齐放进一个黑色塑料袋，并交给每组第一个成员； （2）领导者播放欢快音乐，随着音乐律动成员们开始传递塑料袋； （3）在传递塑料袋的过程中，每位成员务必用力捏一把塑料袋中的水果才能往下传； （4）当音乐停止时，塑料袋就停止传递，主持人请出拿着塑料袋的那个同学，请他当众吃一小口袋里的水果，然后询问同学们尝到的水果和平时吃的哪种更好吃。 | 10min |
| 环保清单 | 知晓日常环保行为，树立日常环保意识 | （1）每个成员列举日常生活中可以实施的环保行为，越具体越细小越好（如不使用一次性筷子、不践踏草坪、随手关灯等），至少20项，越多越好； （2）小组内分享，组长汇总出本组的环保清单； （3）团队分享。 | 20min |
| 环保卫士 | 引导大学生在日常生活中积极践行环保 | 分享感悟：   （1）活动开始前的想法是什么？ （2）实践过程中有什么感受？ （3）活动结束时有何感悟？ | 20min |
| 冥想放松 | 放松身心，结束活动 | （1）分享感受； （2）全体成员跟随音乐舒缓身心。 | 10min |

第六单元：依依惜离别

日期：2023年11月20日	时间：18:30—19:30/19:40—20:40

地点：教育学院团体辅导室	参与人员：17（14）名成员和1名带领者

| 活动主题 | 团体结束：依依惜离别 | | |
| --- | --- | --- | --- |
| 活动目标 | 1. 增强成员间的沟通，增进彼此感情； 2. 强化团体活动效果，产生迁移。 | | |
| 活动准备 | 正念冥想音乐、祝福卡片、签字笔、环保承诺书 | | |
| 活动名称 | 活动目标 | 活动内容 | 时间 |
| 正念冥想 | 放松身心 | 全体成员跟随音乐进行正念冥想。 | 10min |
| 忆往昔 | 总结回顾之前的内容 | （1）小组内成员回顾总结收获，小组长汇总； （2）团体分享； （3）签署环保承诺书。 | 20min |
| 送祝福 | 积极赋义离别情绪 | （1）写下祝福卡片，对象可以是团队，也可以是个人； （2）随机发放，交流卡片内容。 | 20min |
| 惜离别 | 总结感受，结束团体 | 团队带领者总结，结束团体。 | 10min |
